# Neogene fluvial landscape evolution in the hyperarid core of the Atacama Desert

**DOI:** 10.1038/s41598-018-32339-9

**Published:** 2018-09-17

**Authors:** Benedikt Ritter, Finlay M. Stuart, Steven A. Binnie, Axel Gerdes, Volker Wennrich, Tibor J. Dunai

**Affiliations:** 1 Institute of Geology & Mineralogy, University of Cologne, Cologne, Germany; 2 Isotope Geosciences Unit, Scottish Universities Environmental Research Centre, East Kilbride, UK; 3 Institute of Geosciences, Goethe-University Frankfurt, Frankfurt, Germany

## Abstract

Dating of extensive alluvial fan surfaces and fluvial features in the hyperarid core of the Atacama Desert, Chile, using cosmogenic nuclides provides unrivalled insights about the onset and variability of aridity. The predominantly hyperarid conditions help to preserve the traces of episodic climatic and/or slow tectonic change. Utilizing single clast exposure dating with cosmogenic ^10^Be and ^21^Ne, we determine the termination of episodes of enhanced fluvial erosion and deposition occurring at ~19, ~14, ~9.5 Ma; large scale fluvial modification of the landscape had ceased by ~2–3 Ma. The presence of clasts that record pre-Miocene exposure ages (~28 Ma and ~34 Ma) require stagnant landscape development during the Oligocene. Our data implies an early onset of (hyper-) aridity in the core region of the Atacama Desert, interrupted by wetter but probably still arid periods. The apparent conflict with interpretation that favour a later onset of (hyper-) aridity can be reconciled when the climatic gradients within the Atacama Desert are considered.

## Introduction

**Figure 1:**
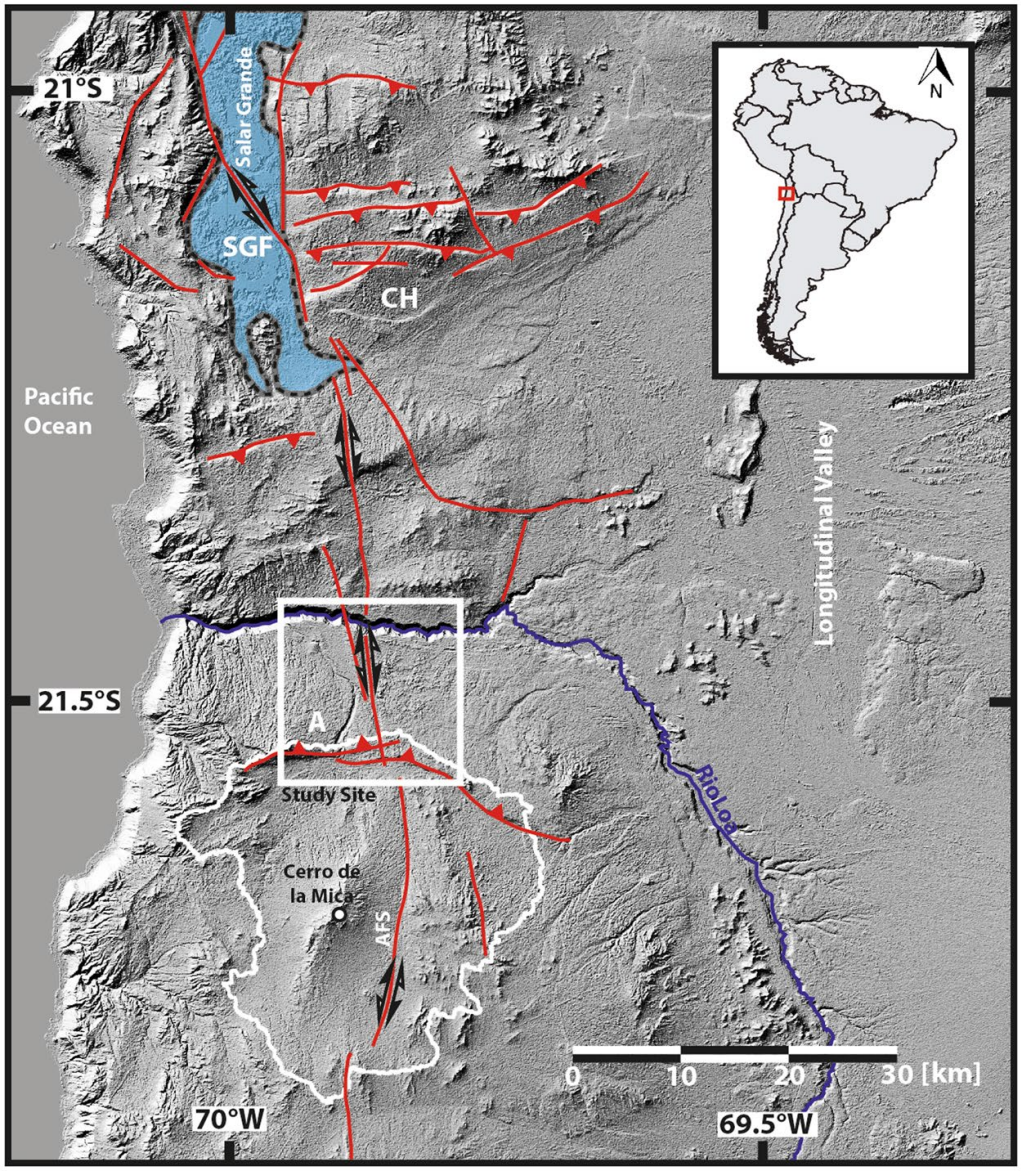
Hillshade image based on Aster GDEM data (30 m resolution, produced using ArcGIS 10.5.1). The study area (white square) is bound toward the north by the perennial Río Loa and to the south by the display source area (white stippled line). Red lines indicate major tectonic fault systems. The enorheic basin that once supplied the sediments investigated in this study is marked with the stippled white line. SGF = Salar Grande Fault, CH = Chuculay Fault, A = Adamito Fault^24,31,92^, this study.

The Atacama Desert of northern Chile is one of the driest places on Earth, with an extreme hyperarid core (Coastal Cordillera & Central Depression between 19° and 22°S), receiving less than 2 mm/yr modern precipitation^1,2^. Subtropical atmospheric subsidence^3^, and the temperature inversion due to coastal upwelling of cold waters of the Peru-Chile Current^1^ have led to hyperarid conditions. The Andes Mountains to the east (Fig. 1) cast a rainshadow over the Atacama Desert as moisture originating from the Atlantic becomes orographically elevated, causing precipitation on the eastern Andean flank and a relative absence westwards^4^. These effects, coupled with high evaporation rates in most areas of the Atacama Desert strengthen hyperarid conditions^4,5^. While the main factors controlling hyperaridity in the Atacama Desert are established, the onset and permanence of hyperaridity remain a matter of debate^6–17^.

The nearly stable position of the South American continent over the least 150 Ma^18^ and the establishment of the Peru-Chile Current system at around 50 Ma^19^ support the notion that predominantly arid conditions persisted since the early Miocene^9^ or potentially earlier^18^. Secular changes of the global climate system during the Cenozoic^20^, led to punctuations of the prevailing arid climate in the Atacama Desert by pluvial phases e.g.^9,10,12,21,22^.

The absence of fluvial erosion means that long-term tectonic activity is a prominent control on landscape evolution in the hyperarid core of the Atacama Desert. Between 19°S and 21.4°S E-W reverse faults^23^ play a major role in generating relief. The resulting scarps, commonly at the angle of repose (35–40°) and devoid of signs of fluvial incision, are up to 300 m high and have formed since at least the Mid-Miocene >15 Ma^24^. The slow vertical movement of these reverse faults (<20 m/Myr) has truncated fluvial channels and blocked drainage^24^. The Atacama Fault System (AFS), a system of primarily trench-parallel strike-slip faults in the area around the Río Loa (Salar Grande Segment)^23^ is a subtler agent of surface modification, largely in the form of horizontal displacement (at unknown rates) and moderate (<50 m) vertical uplift along flower structures. The AFS was active throughout the Cenozoic^23,25^; the topographically subtle surface expressions of its trace are preserved throughout the hyperarid core of the Atacama Desert.

In the present study, we exploit the interplay between slow fault displacement and intermittent fluvial activity to delineate the timing of less-arid periods interrupting the long-term background hyperaridity. We use multiple single-clast exposure ages to determine the timing of tectonically fossilized depositional surfaces, and to detect possible pre-exposure of clasts and/or post-depositional erosion. Our results indicate an Oligocene onset of aridity in the core of the Atacama Desert, with interspersed episodes of enhanced fluvial activity in the Miocene and Pliocene. Preservation of Early Miocene alluvial fan surfaces indicate a predominantly (hyper-) arid climate conditions since the Early Miocene.

## Geological Setting

The central Atacama Desert is located in the fore-arc region of the Central Andes of northern Chile. The Andes mountain orogeny is a consequence of the subduction of the oceanic Nazca plate under the continental South American plate since the Cretaceous^23^. The Peru-Chile trench is located just 70–50 km offshore^23^. The study site is situated in the Coastal Cordillera (Fig. 1), an eroded Jurassic magmatic arc, which consists of a series of Early Cretaceous extensional basins, filled up with Late Oligocene to Early Miocene volcanoclastic sediments^26^. The Coastal Cordillera reaches elevations of between 1000–1800 m, with its westwards extent limited by a coastal cliff that reaches 1000 m above current sea-level and its eastward extend demarcated by the sedimentary infill of the Central Depression. The development of the Oligocene-Miocene (~20 Ma) coastal Tarapaca pediplain coincided with the end of sedimentation in the Coastal Cordillera and the onset of hyperaridity in the Atacama region e.g.^27,28^. Since the formation of this erosional surface, the Coastal Cordillera has experienced tectonic activity; that has produced as much as 300 m of local relief ^24^. The stress field of the slightly oblique plate subduction^29^ gave rise to reverse faults, orthogonal to the plate boundary and approximately parallel to the convergence direction^23^. The resulting exposed fault scarps occur between 19° and 21.6°S in the Costal Cordillera^23^. ^21^Ne exposure ages of planation surfaces east of the Salar Grande (Chuculay Fault system), bisected by these reverse faults, range between 15 and 24 Ma^24^, while a truncated paleo-channel gives an age of 4 Ma^24^. The ages of the planation surfaces place an upper limit for the onset of reverse faulting at the corresponding locations; the age of the truncated paleo-channel indicates the faulting was active after 4 Ma^24^.

## Study Area

**Figure 2:**
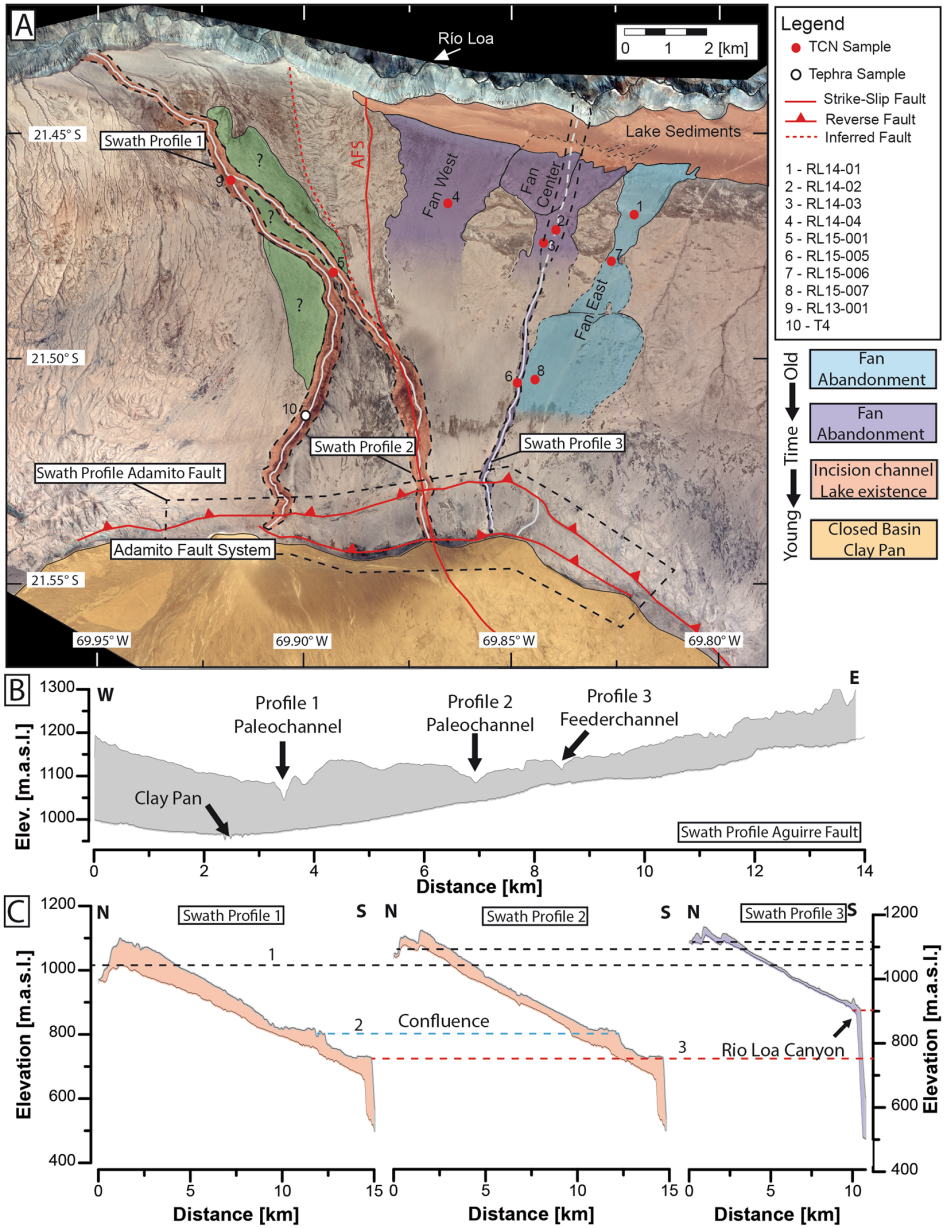
(**A**) Pléiades 1B Multi-Spectral Image of the study area. Red dots indicate locations of samples taken for *in situ* cosmogenic nuclide determinations, and the white dot is the location of the tephra sample. Red lines indicate mapped fault systems (this study), the dashed red line indicates an inferred splay fault. Colour shading provides the relative surface age from geomorphological evidence. No reasonable relative age estimation could be achieved for the green area, based on field and satellite observation. White lines mark major fluvial channels and channel remnants. The areas bound by black dashed lines are used for swath profiles (ArcGIS 10.5.1) of the Adamito fault (**B**) and for Channel swath profiles (**C**) of the three major S-N flowing channels. Black dashed lines (1) in the channel profiles (**C**) mark the elevation of the vertex of the corresponding channels. Blue dashed line (2) marks the confluence area of the two paleo-channel to the east. The red dashed line (3) indicates the elevation of the Río Loa canyon top. Note that these elevations decrease from east to west, here taken as evidence for tectonic tilting of the area and relocation of drainage from east to west.

**Figure 3:**
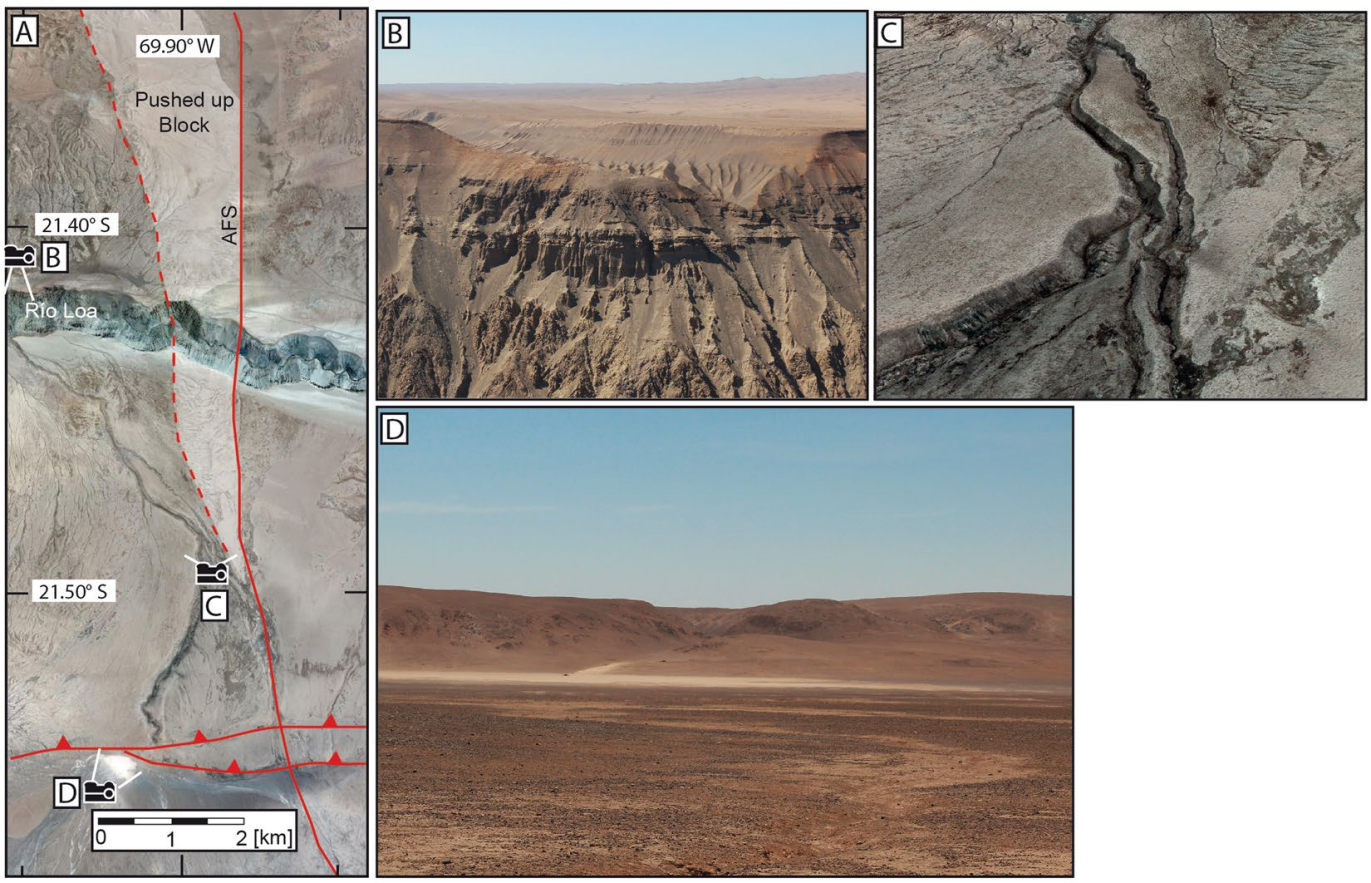
(**A**) Google Earth image (Image data: ©2018 CNES/Airbus & Digital Globe, image recoding 11/7/2014) highlighting the course of the splay fault and uplifted tectonic block. (**B**) Photograph from the northern rim of the Río Loa towards the hanging outflow of the main, westernmost paleo channel (made by B. Ritter). (**C**) Pléiades 1B Pansharped Multi-Spectral 3D image (ArcScene 10.5.1) showing an oblique view of the paleo-channel upstream of the confluence. Except for one tributary channel, the lack of incision of the surrounding flat areas in response to incision of the main channel and generally the smooth valley flanks, points to a predominantly hyper-arid climate since at least the incision of the main channel. (**D**) Photograph (made by B. Ritter) looking north to the endorheic clay pan that was created by the activity of the Adamito thrust fault, blocking its drainage. The ~110 m high ridge in the background is the scarp of the Adamito fault. The depression in the ridge is the wind gap of the main, westernmost paleo channel. The vertex in the wind gap is presently ~50 m above the surface of the clay pan.

The study area is bound to the north by the Río Loa canyon and in the south by the extensions of the paleo catchment (Fig. 1). The main geomorphological features of the study area are, (i) the Atacama and the Adamito fault system (ii) an alluvial fan system east of the Atacama fault (Fig. 2A), with three distinct alluvial fans and feeder channels; and (iii) a system of paleo-channels lying to the west of the Atacama fault system (Fig. 2A). The Adamito fault system^30^ (Fig. 1) bisects the study area. This fault is also named Aguirre Fault^31^, however in accordance with the initial description we will use the name Adamito fault. Prior its truncation by the Adamito fault system, the source area of the alluvial fans (i.e. drainage area above apex^32^) encompassed the Cerro de la Mica (1817 m elevation) and surrounding areas up to 25 km further south (~560 km² - stippled white line Fig. 1), which is now part of an endorheic basin containing a clay pan (Figs 2A and 3D). Based on modern elevation topography, we assume a flow direction from south to north. After the onset of uplift along the Adamito fault the fans’ apex was at or north of the fault. Prior to fault movement the apex of the eastern-most fan might have been south of the fault; however, we found no indication for the latter.

The Adamito (Aguirre) fault system is the southernmost reverse fault system in the Coastal Cordillera^23^. It forms a steep scarp, partially modified by gravitational slumping (Fig. 3D). The cumulative vertical offset created by the set of two faults (Adamito and Atacama) is up to 130 m; truncating the paleo-drainages. The three paleo-drainages are identified by the continuity of incised-channel forms, yet the paleo-thalwegs have an intermediate high-elevation position, referred to as the vertex, which now divide the incised channels into a south-draining and a north draining region.

The elevation of the dissected channels, measured at the highest present day water-divide, decreases from 1090 m for the easternmost channel (Fig. 2B,C) to 1000 m for the westernmost paleo-channel with a value of 1050 m for the middle channel (Fig. 2B,C). Assuming that the kinematics of fault movement did not change during the incision of the paleo-drainage, it follows that the ages of the paleo-drainage decrease moving from east to west.

The elongated N-S extending trench-parallel Atacama fault zone runs through the study site (Figs. 1, 2). The AFS extends north to the Salar Grande, where it causes vertical displacement of up to 50 m^23,25,33^. In conjunction with an inferred splay fault (sub-parallel secondary fault of the AFS) close to the Río Loa (Figs 2A and 3A), the AFS creates a local topographic high, about 20–30 m higher than the alluvial fan surface to the east and up to 90 m higher than the west. No visible vertical offset could be observed at the Río Loa canyon walls, as hillslopes are largely covered by debris. The vertical offset is evident from incised fluvial channels in the uplifted block north of the Río Loa, (Supplementary Fig. 5). Surface deposits obscure signs of recent horizontal displacement.

## Results

### Alluvial Fans

The alluvial fan system consists of three distinct alluvial fan surfaces (Fig. 2A). Their outline can be identified on satellite imagery (Google earth, Pleiades used here, Fig. 2, and supplementary data set) and on the eastern-most fan indicative surface structures (sediment lobes in the direction of paleo-flow) are visible. Due to extensive gypsum soil cover the fan surfaces are less conspicuous on the ground, on first sight resembling any near-horizontal surface in the dry core of the Atacama. Feeder channels and lateral fan boundaries, however, are discernible on the ground. Identification and selection of sampling sites was always guided by satellite imagery.

The fan surfaces are dipping to the north, (0.9°–1.6°), with decreasing slopes from east to west (eastern fan 1.46°, central fan 1.3°, western fan 0.9°). The source area of the depositional surfaces can be related to the endorheic catchment to the south (Fig. 1). This assumption is based on the modern flow direction of the closed catchment and the direction of paleo-channels and feeder channel of alluvial fans north of the Adamito fault. Distal fan surfaces near the Río Loa canyon are buried by lacustrine sediments related to lake episodes in the Central Depression (diatom-rich sediments and evaporitic remnants of the Quillagua (Pliocene) and Soledad Fm (Plio-Pleistocene)^13,34^). Cross-cutting relationships of the fans are partially obscured by the lake deposits; however, the eastern-most margin of the central fan can be inferred as cutting into the eastern fan (Fig. 2A black stippled lines and Fig. 6D supplementary data set). The maximum sediment thickness of alluvial and lacustrine strata is approximately 85 m, as exposed in outcrops along the Río Loa canyon. The lacustrine deposits occur exclusively east of the topographic high formed by the AFS (Fig. 2A). The distal portions of the alluvial fans resemble an inverted landscape, whereby lateral fan boundaries commonly end abruptly and narrow, channel-like sediment lobes are several meters (up to 8 m) above their surroundings. The steep lateral fan boundaries are probably enhanced by differential erosion of (coarse) fan-sediment covered areas and areas that lack this cover. Areas adjacent to the flanks are commonly endorheic deflation hollows. Strong westerly anabatic winds, associated with the diurnal heating of the Altiplano to the East^35,36^, are the likely cause of deflation of neighbouring – presumably (since they are now missing) finer grained sedimentary rocks.

The surfaces of the eastern and central fan are essentially flat, with long-wavelength (10–100 m scale) topography variations on the cm-scale. The surfaces are covered by gypsum-rich soils that typically comprise a 10–30 cm powdery, locally friable, layer chuca, senu Ericksen^37^, underlain by a moderately to firmly cemented sediment layer costra, senu Ericksen^37^. Due to the cementation of this layer its depth could not be determined in this study; usually it is 0.5 to 2 m thick^37^. The gypsum accumulations in the soils are due to atmospheric deposition of calcium sulphate^16,38,39^ onto the alluvial fan surfaces. Following the ‘born at the surface’ model of Wells *et al*.^40^ stones of a desert pavement remain at the surface on an accretionary mantle of soil-modified dust and are successively lifted from the bedrock/original sediment surface; this process has been confirmed as being operational in hyper-arid soils of the Atacama^38^ and allows the continuous exposure of clasts on the surface of the alluvial fans. Any modifications of the soil-surface by deflation or gentle fluvial erosion would keep clasts at the surface.

The surfaces of the eastern and central fan were devoid of any indication of recent runoff able to erode the gypsum soil. The observed polygonal pedogenic surface patterns do not relate to gradient and are not used for runoff. The surface of the westernmost fan has medium-wavelength (horizontal ~10 m scale) variations in the micro-topography on the dm-scale. Traces of recent (Quaternary) runoff are ubiquitous, resembling shallow braided channel systems. Powdery gypsum-rich soils are largely absent, possible remnants (each <1 m^2^) remain in the centre of some braids.

Rock fragments are rare on the fan surfaces (on average less than 1 clast per 25 m^2^) and consist predominantly of vein-quartz (>95%). Some clasts retain fluvial rounding, most have angular shapes, which derive from ‘kernsprung’ (insolation weathering). On the central and eastern fan, angular fragments commonly occur in clusters on the surface, due to ‘kernsprung’ of former larger clasts (vein quartz – polymineral quartz would already have disintegrated and eroded) and the limited amount of observed dispersion of these fragments (mostly <2 m) indicates surface stability and limited diffusive transport. Advective fluvial transport would have dispersed the clasts further and destroy clusters. Sharp edges of quartz clasts that were destroyed by ‘kernsprung’ limit aeolian modification/erosion to less than 1 mm. We found no evidence of windkanter-like modification of quartz clasts (ventifacts), which is probably due to the dominance of gypsum surface materials in the study area and the rarity of quartz in the size fractions transported by wind (i.e. sand and smaller).

Vein-quartz is a trace constituent of the rocks in the feeder (paleo-) catchment (Jurassic granitoids and Devonian/Carboniferous metasediments^30^). As such the vein-quartz constitute a lag deposit, with fine-grained and/or polymineralic rocks removed by physical erosion (salt-weathering, abrasion) and chemical weathering^41^. The study area experiences regular sea fog^42^ which has probably enhanced chemical weathering. Modern analogues for the channels that would have shed sediments onto the paleo-fans exist as 10–15 m wide active channels, presently draining towards the clay pan adjacent to the Adamito fault (Fig. 2A). In these channels, granitoid and metasediment clasts dominate; vein quartz clasts, equivalent to those found on the fan surface, constitute less than <1% of clasts found in the large channels. The feeder channel of the central fan is well preserved and deeply (~12 m) incised into older fan-deposits. Several faint feeder channels are visible near the fault scarp (Fig. 2A white lines).

### Paleo channel System

A paleo channel system with one main channel (~16 km length) lies to the west of the alluvial fans and one lateral tributary (~11 km length) to the east. (Figs 2A and 3C). The formation of the eastern tributary channel coincides with the topography along the Atacama fault zone and its irregular morphology is affected by fault activity and slumping. Both paleo-channels were deflected north of the Adamito fault from a north to northwest direction by a topographic high, presumably developed by the interaction of the AFS and an adjacent splay fault. To the west of the Atacama fault zone, both channels have smooth V- shaped cross-sections, which are occasionally modified by gravitational mass-movement on their steep slopes. In the central section, (ca. 5 km north of the Adamito fault and south of the confluence of the central and tributary channel) fluvial modification of channel hillslopes is limited to isolated patches with rill-erosion. The dominant hill-slope process is gravitational movement. Hillslopes have prominent cracks at a steep angle to the gradient. Minor cracks are gravitationally modified soil polygons. The morphology of the cracks is not fluvially enhanced. The channel downstream of the confluence shows signs of fluvial incision, resembling badland erosion near to the confluence with the Río Loa canyon. At the confluence with the Río Loa the paleo-channel terminates as a hanging-valley, ~450 m above the channel bed of the perennial Río Loa (Fig. 3B).

The two paleo-channels incise 40–60 m into the surrounding gypsum-soil covered flats, with the incised depth increasing downstream. There is no indication of any sedimentation by the paleo-channels onto the adjacent flats, prior to their incision. The paleo-channels run straight for most of their course, though some portions have noticeable sinuosity. For the first 1.5 km just north of the Adamito fault zone the main paleo-channel has a sinuosity of ~1.3; a short segment (600 m) in the lower reaches of the tributary channel has a sinuosity of ~1.15 (Fig. 2A). Portions of the main channel, particularly on the western side of the channel, are covered by up to 1 m thick pure pristine tephra (i.e. without xenolithic/extraneous lithic fragments) with a thin surficial coating (<5 mm) of friable gypsum crust.

### Zircon U/Pb dating

Single zircon tephra ages (n = 62) derived by U/Pb dating range from 438 to 0.86 Ma (see Supplementary Table 3). U/Pb zircon ages reflect different degrees of contamination by zircons derived from pre-eruption country-rock and reworking. The youngest cluster of zircons within 2σ represents the eruption age. The age spectrum is dominated by zircon ages around 1 Ma. The oldest group, less numerous, represents xenocrystic grains which could result from assimilation of local bedrock material during magma ascent or eruption (zircon U/Pb ages older than 20 Ma). Taking the cluster of youngest zircon ages as indication for the eruption ages, is based on the assumption that young zircons are unlikely to have experienced significant Pb loss, which would result in anomalously young ages^43^. Based on published eruption data from volcanic complexes within the Atacama Desert, the zircon ages of the tephra can be linked to the latest eruption of the Purico Complex, with an eruption age of 0.98 ± 0.03 Ma (2σ) (Ar/Ar-dating on biotite^44^) or the Tatio ignimbrite 0.703 ± 0.010 Ma (2σ) (Ar/Ar-dating on sanidine^45^). Both belong to the Altiplano Puna Volcanic Complex^46^. Due to a potential pre-eruptive crystallization history, and the early crystallization duration of zircons^46^, a direct assignment to one of those eruptions is not possible. The pre-eruptive evolution of the Purica centre lasted 290 ka^46^. The purity of the sampled ash, i.e. the absence of extraneous lithic fragments, precludes fluvial reworking since its deposition in the paleo-channel. This implies that the paleo-channel has not carried surface run off since at latest 0.7–1 Ma.

### Cosmogenic exposure ages

#### ^21^Ne exposure ages

**Figure 4:**
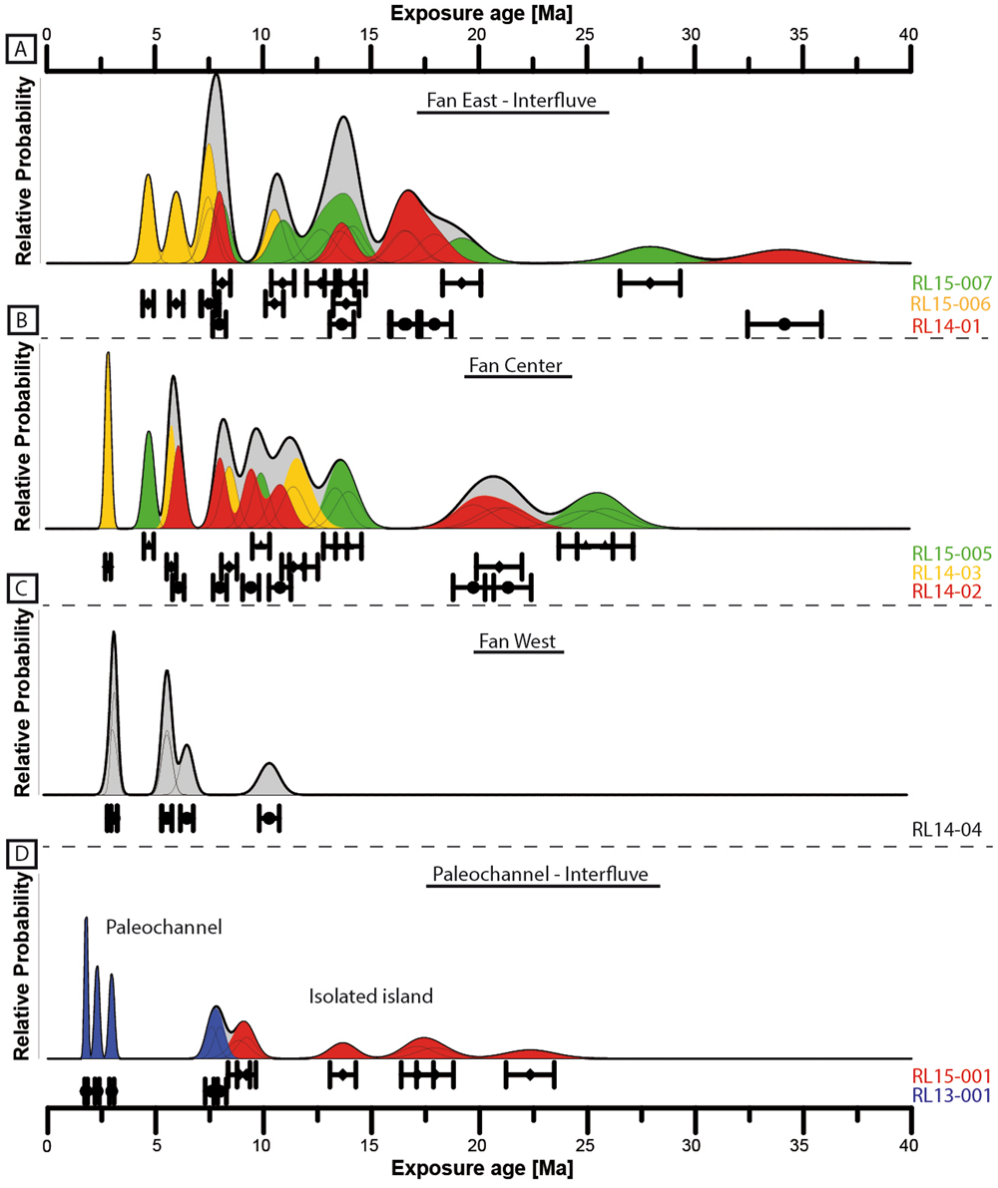
Cumulative probability density plots of ^21^Ne (±1σ) single-clast exposure ages from the study area. Sub-plots display the data for each fan/channel system. Grey areas reflect the cumulative distribution of all samples of one system, coloured areas reveal cumulative probability of individual sampling sites within a system. The data is also presented as points and error bars (±σ) arranged in lines for individual sampling location (corresponding sample names are shown at the left; the text colours reflect the colour of the density plot) below the density plots. The locations of the systems and sampling sites area shown in Fig. 2.

Cosmogenic ^21^Ne concentrations have been measured in 53 samples from 9 sample sites (Fig. 2A, Supplementary data Table 1). Exposure ages (^21^Ne) range from ~2 Ma to up to ~34 Ma (Fig. 4). The majority of ages (>80%) are younger than 15 Ma and the fan surfaces yield no age younger than 3 Ma. In the following three or more ages with overlapping uncertainty envelopes are used to delineate a group of ages.

On aggregate, the ages of the three samples from the eastern fan system (RL15–006 & 7; RL14-01) have one negatively skewed group of ages between ~16.5–19.5 Ma and one symmetrical group of ages between ~13–14.5 Ma (9 of 18 ages; Fig. 4A, Supplementary data Table 1), a third group around ~7.5–8 Ma (3 of 18 ages), and individual ages at ~4.7, ~6, ~11, ~28, and ~34 Ma. Individual, old, clasts (>20 Ma) occur in two of the three samples (RL14-01, RL15-007). The highest exposure age (~34 Ma) is amongst the oldest reported for the Atacama Desert^9,10,24^.

In total the ages of the three samples from the central fan system (RL15-005; RL14-02 and 3) have a main negatively skewed group of ages between ~14 and 8 Ma (9 of 18 ages; Fig. 4B, Supplementary data Table 1). Additional groups occur between 19–25 Ma (5 ages), two ages at ~6 Ma, and individual ages at ~5 Ma and ~3 Ma.

The six ages of the westernmost fan surface (RL14-04) range between ~10.5 and ~3 Ma, with a group of three ages around ~5.5–6.5 Ma (Fig. 4C).

The samples from the surface that was isolated by the incision of the two branches of the paleo-channel system in the west (RL15-001), range between Early to Late Miocene (Fig. 4D), with individual ages at ~22, ~17.5 (n = 2), ~14, and ~9 Ma (n = 2). The samples taken from the incised paleo-channel (RL13-001) show similar ages at ~8 Ma (n = 2), and the youngest being ~2 Ma (n = 2).

#### Cosmogenic ^10^Be exposure ages

Concentrations of *in situ* produced cosmogenic ^10^Be were determined for six quartz clasts (Supplementary data Table 2), four from the easternmost (RL14-001) and two from the central fan system (RL14-02). The ^21^Ne ages of the RL14-001 clasts range between ~14–18 Ma, those of the RL14-02 clasts between ~8–10 Ma. Within typical measuring precision of several percent, cosmogenic ^10^Be concentrations reach saturation (secular equilibrium) after around two to three half-lives (^10^Be 1.36 ± 0.07 Ma^47^), when the rate of ^10^Be lost via radioactive decay and the rate of cosmogenic ^10^Be production become similar. In such cases, the ^10^Be concentration becomes time invariant and no further information can be obtained from the sample, other than it has been exposed for the minimum time required to reach saturation^48^. All but one sample are at, or near, saturation with respect to ^10^Be, indicating a continuous exposure at the surface over at least the last 4 Ma, approximately. One sample (RL14-02 clast e) yields a ^10^Be exposure age of 1.8 ± 0.2 Ma. The saturated (or near-saturation) samples correspond to high ^21^Ne ages; the single age of 1.8 ± 0.2 Ma (RL14-02 clast e) indicates the recent exhumation of this particular clast from a temporarily (partially) shielded position.

### Interpretation

#### Cosmogenic exposure ages of depositional surfaces

*In situ* cosmogenic nuclide concentrations are now widely used to determine the timing of sediment deposition in the Atacama Desert^6,8–10,21,24,28,49,50^. Accurate deposition ages require that the accumulation of cosmogenic nuclides during erosion and transport prior to deposition are negligible, or can be determined, and the effect of post-depositional erosion/exhumation can be identified. Significant pre-exposure leads to erroneously old deposition ages, whereas post-depositional erosion/exhumation lowers the age of deposition^48^. Multiple single clast ages from a depositional surface can be used to test these effects^51^. Tight groups of ages are expected if the clasts were previously unexposed material with short transport times relative to deposition time. Erosion of low-angle surfaces will result in the dispersal to younger ages, as the resulting lag-deposits contain material exhumed from various depths of shielding. In the absence of pre-exposure, the highest ages obtained on such a surface are the best estimate for the minimum deposition age.

Pre-exposure, however, can be significant, particularly in (hyper-) arid environments that are mosaics of ancient surfaces. In the core of the Atacama Desert, Early- to Mid-Miocene surfaces are widespread and even traces of older, Oligocene surface material have been reported^9,10,21,24,28^.

In this setting there are three main scenarios during which pre-exposure occurs: (i) accumulation of cosmogenic nuclides during exhumation of material in the source region, (ii) during protracted sediment transport; and (iii), a supply of sediment from a formerly stable, older sedimentary surface. Scenario (iii) would require clasts to be deposited on the fan surfaces with a significant and similar pre-exposure signal, which is unlikely given the stochastic nature of erosion and transport processes that tend to add variable amounts of inheritance to the age-signal^52,53^. Clustered, non-random, pre-exposure signals would be possible only where clast source areas were old, stable surfaces. Proximity of such a hypothetical old source (similar to the recent (hyper)-arid core of the Atacama Desert) surface to the sedimentary deposit to be dated is required to prevent dilution (beyond recognition) of the clustered pre-exposure signal, by ‘normal’ randomly pre-exposed material.

We observe differing modes of pre-exposure in our data set: the common random pre-exposure and likely traces of clustered pre-exposure. Several ages of the eastern fan deposits (samples RL15-007, RL14-01) show signs of significant pre-exposure, with two individual ages ~7 and ~15 Myr older than the next oldest age in the main age group (~19 Ma, Fig. 4A). These pre-exposure signals signify that at the time of deposition of the eastern alluvial fan, remnants of an Early Miocene/Oligocene landscape existed in the source area south of the Adamito fault system. The minimum deposition age of the eastern fan system is the oldest age in the main group, ~19 Ma. The ages <19 Ma reflect post depositional modification of the fan surface. The youngest ^21^Ne age of ~5 Ma and the saturated ^10^Be samples (Supplementary data Table 2) indicate that significant surface modification on this fan ended prior to ~5 Ma.

Several samples from the central fan system also contain pre-exposure age signals. The central fan-system is stratigraphically younger than the eastern system - it cuts the eastern fan near the Río Loa (Fig. 2A) - but yields five ages older than the main group of the eastern fan (Fig. 4A,B). The upper (older) age limit of the main age group of the central fan is ~14 Ma. The five older ages show signs of clustering, three are overlapping ages around ~19–21.5 Ma, and two at ~25 and ~26 Ma. Since the feeder channel of the central fan system incised into the substrate of the older eastern fan, it is evident that this substrate must have been eroded and transported to the surface of central fan .The initial incision of the feeder channel, possibly laterally less well defined at its onset, is a possible source of the clasts carrying the Early- to Mid-Miocene ages. We cannot rule out that the pre-exposed clast came from the catchment south of the Adamito fault system; however, this is unlikely given the requirement for a proximal source to preserve clustered pre-exposure. Younger ages reflect post depositional modification of the fan surface. The youngest ^21^Ne age,~3 Ma and the ^10^Be age of ~1.8 Ma indicate that surface modification on this fan continued into the Quaternary.

The ages of the western fan do not cluster and potential pre-exposure cannot be resolved. However, since the maximum age is ~10.5 Ma, a sediment contribution from Early- to Mid-Miocene surfaces can be excluded in this case (Fig. 4C).

#### Chronology of erosion, deposition, tectonic displacement and incision

From the geochronological constraints and the observed field relationships we reconstruct a history of erosion, deposition, tectonic displacement, and incision. From the exposure ages obtained for alluvial fan surfaces, it is striking that one in six clasts analysed (7 of 43) have an Early Miocene or older age exposure age, with a conspicuous group (n = 5) of ages bracketing the Oligocene/Miocene boundary (Fig. 4). It is likely that the source area of the sediments contained areas that had been stable since the Oligocene/Miocene transition. The interfluve preserved between the branches of the paleo-valley (sampling site RL15-001, Fig. 3C) may be an analogue of these past source regions, since it preserves ages up to the earliest Miocene.

The oldest ages, ~28 and ~34 Ma, indicate that the denudation of the source regions prior to the deposition of the fans (i.e. prior to Early Miocene) was insufficient to remove all surface material of a pre-existing landscape. These old clasts had been at or close to the surface for 9 to 15 Myr, or longer if intermittent burial is assumed. Possible source areas could not have been much higher (the summit of the Cerro de Mica is 800 m higher, Fig. 1), thus surface exposure cannot be reduced by much by postulating periods of early exposure at higher altitudes. We conclude that the time-integrated denudation in the source region was low throughout the Oligocene.

**Figure 5:**
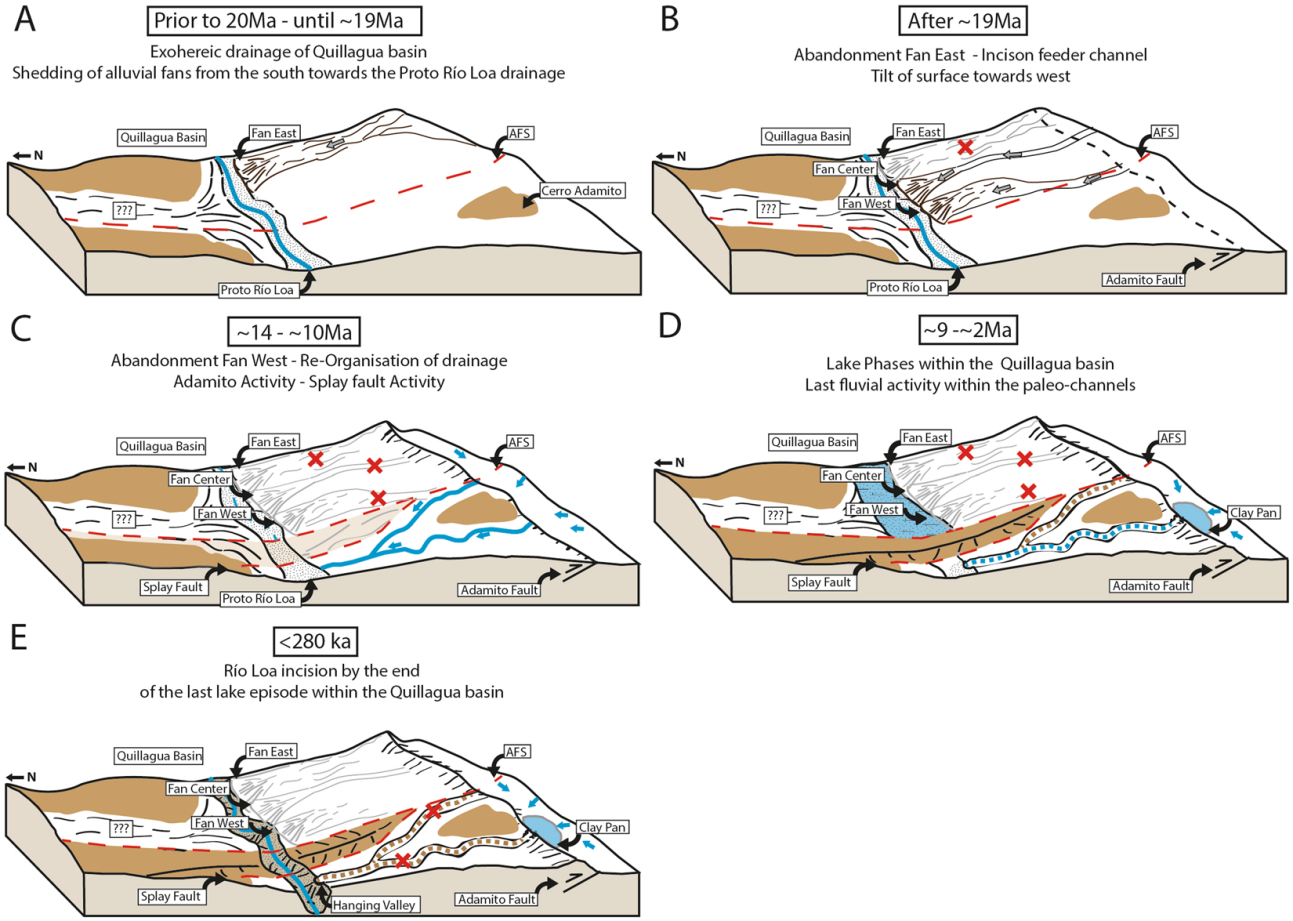
Sketches of the geologic and geomorphologic evolution of the study area through time, based cosmogenic nuclide exposure data (this study).

Deposition of the easternmost fan north of the Adamito fault terminated in the Early Miocene (~19 Ma), based on the oldest ages of the main group from the eastern fan (Figs 4A and 5A). The absence of any visible feeder channel on the main alluvial fan surface may indicate that this fan system became inactive due to a slow backfilling, rather than by tectonic truncation. Remnants of a former feeder channel are just visible at the Adamito fault scarp, as a small incised channel (Fig. 2A, white line trending only across the Adamito fault). Subsequent deposition switched to the central fan, which terminated during the late Mid Miocene (~14 Ma), based on the oldest ^21^Ne ages for the central fan (Figs 4B and 5B). The central fan has a well-developed feeder channel, indicating that the truncation of the drainage rather occurred due to tectonic uplift along the Adamito fault, which had commenced by this time *and* had outpaced fluvial incision. Lastly, sediments of the western fan were deposited during the Late Miocene (~10.5–9.0 Ma), from the age range defined by the oldest clast on the fan and the onset of incision of the paleo-channels to the west (Figs 4C and 5C). A likely candidate for the feeder channel of the western fan is the eastern channel of the deeply incised paleo-channel system straddling the Atacama fault zone (Fig. 2A). This possibility, however, could not be substantiated in the field, due to extensive surface cover by dust Chuca^37^. Fan deposition to the north of the Adamito fault terminated with the incision of the large paleo-channels straddling west of the AFS, during the Late Miocene (≤~9.0 Ma), indicated by the youngest age on interfluve and oldest age of clasts the in channel (Figs 4D and 5C).

Lake sediments deposited on alluvial fan surfaces constrain a minimum age for the deposition of the alluvial fans to be earlier than 5.54/5.36 Ma^13^). Based on our field observations, sediments of the Quillagua and Soledad Formation lie on top of the distal portions of the alluvial fans. The first appearance of a closed basin within the Quillagua basin occurred around 8.8 Ma (Hilaricos Unit^13^). Thus, the course of a proto-Río Loa through the Coastal Cordillera was blocked at or prior to 8.8 Ma. Fluvial sediments of a ‘Proto-Río Loa’ system are visible on both sides of the AFS (Fig. 4 supplementary data set). Likely causes for the shift from an exohereic drainage system to a closed, endorheic, system in the Quillagua basis are the relative uplift of the Coastal Cordillera with respect to the Central Depression and/or a blocking due to the activity and interaction of the AFS and NW-SE occurring splay faults (Fig. 5C,D).

The youngest ages from clasts in the paleo-channel (~2–3 Ma) and the age of the volcanic ash in the channel (0.7–1 Ma) constrain the minimum age for the last fluvial transport in the channel (Fig. 5D). The fact that the main paleo-channel terminates as a hanging valley at the Río Loa canyon, incised into unconsolidated clastic sediments (Fig. 3C), indicates that the incision of the paleo-channel terminated prior to the incision of the Río Loa (Fig. 5E) in the Middle/Upper-Pleistocene (post MIS 10)^54,55^.

Sedimentation in the basin, south of the Adamito Fault, commenced at the *latest* after the termination of fluvial incision and transport in the paleo-channels (see previous paragraph). Since incision of the paleo-channel could have also been driven by spilling of an (ephemeral) paleolake at the position of the present-day clay pan, lacustrine sedimentation south of the Adamito fault may have commenced at any time after ~9 Ma (Fig. 5D). Recent drilling of the clay pan in the course of the CRC 1211 (http://sfb1211.uni-koeln.de/) revealed that at least 50 m of sediment was deposited in the pan since tectonic truncation of the paleo-channel. Cumulative tectonic displacement since ≤9 Ma on the adjacent section of the Adamito fault is at least 150 m, translating into a long-term average rate of vertical tectonic displacement of at least 15 m/Myr.

Similar to soil studies by Wang, *et al*.^38^, major surface modification on the alluvial fans ceased around ~5 Ma, coincident with a major drying of the Atacama Desert. The youngest ages obtained from the fan surfaces (^21^Ne ~3 Ma, n = 3; ^10^Be ~2 Ma n = 1) Fig. 4), indicate that significant fluvial surface modification in the study area terminated around 2–3 Ma. Thereafter, fluvial processes were unable to excavate pebbles from previously shielded positions.

The displacement of the drainage from east to west and the relative chronology is supported by the crosscutting relationship of the central and eastern fans (Fig. 2A), and the decreasing altitude of the wind gaps (i.e. the highest elevation of the fossil drainage channels) from east to west (Fig. 2B,C). Differential uplift along the Adamito fault, tilted the surface to the west and drove the direction of channel diversion (Fig. 2C). The uplift along the Adamito fault, in conjunction with insufficient discharge to allow fluvial incision to keep pace with fault uplift caused progressive abandonment of the fans and subsequent abandonment of the paleo-channel. To address whether temporary, discontinuous uplift or temporal fluctuations in the water availability provided the timing for the diversions and abandonment, and to make statements on the paleoclimate of the study area, we compare our chronology to other records from the region.

A recent compilation of the regional chronostratigraphy of sediments in the Longitudinal Valley in northern Chile, due east of our study area (Fig. 1) identifies regional, abrupt terminations of sedimentary deposition at ~23 Ma, ~19 Ma, ~13 Ma, ~11 Ma, ~7 Ma, and ~3 Ma^28^. These correspond to the timing of aggradational and degradational surfaces in the Coastal Cordillera and the Andean foreslope^21,28^. Hiatuses in the sedimentary record of the Longitudinal Valley are predominantly climate-induced due to intensification of (hyper-) aridity^28^. We note a good agreement with our ages for inferred surface stabilization in the source area at ~23 Ma (the O/M boundary) and terminations of fan deposition at ~19 Ma and ~14 Ma, as well as the termination of fluvial surface modification at ~2–3 Ma. The Late Miocene incision of the paleo-channel (≤9 Ma), is equivalent to the deposition of sedimentary unit 5 of Evenstar, *et al*.^28^. We note that discontinuous tectonic movement is not required to explain the *timing* of hydrological re-arrangement in our study area. However, it remains a possibility. In the following we treat the chronological constraints derived for our study area as synchronous to the regional chronostratigraphy^28^, and thus synchronous with changes in the regional climate.

#### Paleoclimate of the Coastal Cordillera near the dry core of the Atacama

The study area is unique in its position, located in the Coastal Cordillera near the core region of the Atacama (in terms of dryness), with a present-day annual rain precipitation well below 2 mm^1^. The climate-sensitive changes of erosion, deposition, and drainage re-arrangement provide a glimpse into the past climatic conditions. Most climatic reconstructions of the Atacama Desert rely on records that owe their existence to rainfall on the Andean foothills, the Precordillera, or even the Altiplano e.g.,^12,13,17,21,22,28,56–60^ or are located in areas south of 23°S that sometimes receive appreciable, Winter-rainfall e.g.,^61–66^. Currently, the Andean foothills of the Atacama receives 10–100 times more precipitation than the region of this study in the Coastal Cordillera^1^. Thus, while the *timing* of changes in the regional climate are probably synchronous throughout most of the Atacama Desert (see above), the local expression of climatic conditions and surface processes were most likely different, subject to sometimes steep climatic gradients.

The oldest clasts found in our study (~28 and ~34 Ma) are age-equivalent to the Azapa formation of the Longitudinal Valley^28^. Similarly, high ages have been reported for fluvial clasts in the Coastal Cordillera at ~19.5°S (~27 and ~37 Ma^9^). Earlier we have argued that their long surface exposure during transit points to low erosion rates in their source region in the Coastal Cordillera during the Oligocene. During the same period, up to 500 meters of Azapa sediments were deposited in the Longitudinal Valley^67^ in response to Andean uplift and aided by orographic rainfall^28^. Thus, while the Oligocene climate in the proto-Andes was sufficiently wet to allow a significant denudational response to steepening and hillslope gradients, denudation and sediment transport in the Coastal Cordillera appears to have been much more subdued, despite significant local relief of ≤800 m. It is difficult to accurately constrain the climatic conditions that leave pebbles in a fluvial system in transit at or near to the surface for at least 9–15 Myr, though it is likely arid rather than semi-arid, since the latter is prone to accelerated erosion^68^. Aridification at the Oligocene/Miocene boundary, presumably to hyper-arid conditions, brought erosion and deposition in both areas to a temporary standstill^9,28^. This hiatus in sedimentation led to the long-term preservation of the regionally occurring Tarapaca Paleosurface^28,69^, which was formed by erosion of the Coastal Cordillera, the emerging Andes and sedimentation in the Coastal Cordillera to form an Oligocene low-relief surface^28^. Our results and a previous study^9^ indicate that, at least in some areas of the Costal Cordillera significant deposition and erosion terminated as early as the Oligocene/Eocene boundary.

The volume of sediment preserved in the alluvial fans investigated is small (~0.3 km^3^; estimated 60 km^2^ surface area, ~5 m thickness) considering the size of their catchment (~560 km^2^). A time-integrated (from 23 Ma to ~9.5 Myr) catchment-wide average of as little as ~55–60 cm erosion could explain the amount of sediment deposited. Although we cannot quantify the amount of material eroded from the catchment upstream that has not become part of the fan deposit, it appears that average erosion rates throughout the Early and Mid-Miocene could have been well below 1 m/Myr. Again it is difficult to define the climatic conditions; however, a persistent semi-arid climate as has been suggested previously^23^ is probably out of the question. A possible scenario is that arid phases (fan deposition) interrupted a predominantly hyper-arid climate in this portion of the Coastal Cordillera in the Early- to Mid-Miocene.

#### Onset of (hyper-) aridity

**Figure 6:**
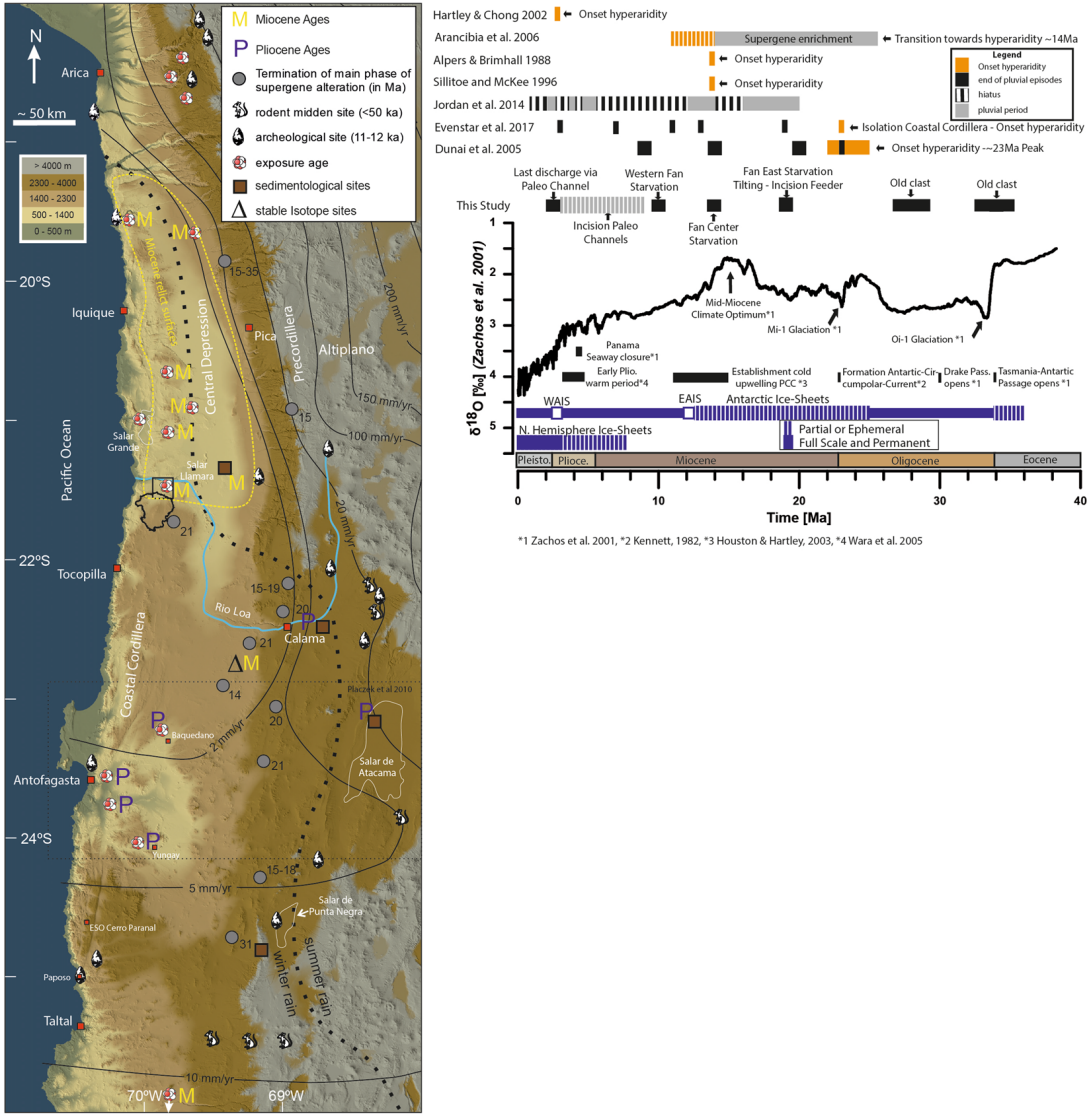
(**A**) Colour shaded digital elevation model (derived from SRTM-data, created using ArcGIS 10.5.1) with isohyets^1^. Dashed black line indicates the border between winter- and summer-rain dominated areas^1^. Sites from the literature are: terminations of phases of supergene enrichment of porphyry ore-deposits^14,93–95^, rodent midden sites^22,62^, earliest archaeological sites^60^, stable isotope studies^96^, sedimentological studies^12,21,97^, exposure ages and erosion rates determined with cosmogenic nuclides^7–10,24,28,49,50,61,97,98^. The stippled yellow outline for Miocene relict surfaces is derived from studies yielding Miocene exposure ages (M) for sediment surfaces^9,10,24,28^ and sedimentological studies^21^. Studies yielding Pliocene ages for the onset of aridity are marked with P. Study site is marked with a black rectangle and drainage catchment in black. (**B**) Global deep-sea oxygen isotope stack based on data from Zachos, *et al*.^20^. Vertical blue bars indicate a qualitative representation of ice volume in each hemisphere relative to the LGM, dashed parts indicate episodes of minimal ice cover (<50%), full bars represent close to maximum ice coverage (>50% of present) from Zachos, *et al*.^20^. Major global tectonic and climatic periods/events are marked. Terminations of periods of landscape modification, in the case of fan and drainage abandonment (this study), and onset of channel incision (this study, dashed grey line) are marked with black bars. The duration of these wetter periods are derived by combing our data with the regional chronostratigraphy^28^ and paleo-environmental reconstructions^21,28^. Black bars indicate reference records from Atacama Desert, which reveal the end of wetter conditions at certain times and places. Reconstructed pluvial phases and hiatuses are marked in grey and black-white dashed lines, respectively^21^. Arancibia, *et al*.^94^ reconstructed a rapid decrease in the frequency of supergene oxidation ages from 14 Ma onwards, with a final cessation at about 9 Ma (dashed orange bars). Alpers and Brimhall^93^ and Sillitoe and McKee^14^ dated the end of supergene enrichment at ~14 Ma. End of supergene oxidation is interpreted to reflect the transition from semi-arid towards hyperarid conditions^99^. Wet phases reconstructed for the Andean fore-slope, due east of our study area, are from Jordan, *et al*.^21^. Regional episodes of deposition and surface stabilization in the Atacama Desert are from Evenstar, *et al*.^28^. The proposed onset of hyper-aridity based on sedimentary deposits within the Calama basin by Hartley and Chong^12^ is marked in orange. TCN exposure ages from the northern Coastal Cordillera, indicating the end of wetter episodes (black) and onset of hyperaridity (orange) are from Dunai, *et al*.^9^.

Whether the onset of (hyper-) aridity in the Atacama Desert is relatively recent Pliocene e.g.,^12,13,18,61,70^ or ancient Miocene or earlier e.g.,^9,10,14,21,28,57^ is subject of ongoing debate (Fig. 6). The differences in interpretations are largely related to the regional distribution of the evidence and on the sensitivity of the proxies used^71^ (Fig. 6). Figure 6 displays a compilation of studies that have estimated the onset of hyper-aridity in the Atacama Desert. The map (Fig. 6) reveals locations of studies in the Atacama Desert and illustrate how they relate to recent precipitation patterns^1^. The additional graph displays age constrains for the onset of hyper-aridity of selected studies, compared to global climate reconstruction based on deep-sea oxygen isotope stack on data from Zachos, *et al*.^20^.

Recent studies, e.g.^21,28,72^ (Fig. 6) demonstrate that the relative regional climatic gradients in the Miocene were not unlike those of today^1,73^. As is found presently, the aridity in the Miocene became more extreme towards the dry core of the Atacama (Coastal Cordillera & Central Depression between 19° and 22°S) from the North^28,72^, and from the East^21^ (Fig. 6). The Andes control the distribution of available moisture on their western flank through a combination of orographic precipitation and deflection of atmospheric circulation in the Summer-rainfall-dominated areas of the Andean foothills^1,4,21,72^. Moisture sources of the Summer-rainfall-dominated areas are located east of the Andes^1,4,21,72,73^.

Winter-rainfall sourced in the Pacific Ocean dominates south of the dry core (Fig. 6)^1,73^. This area has experienced a dynamic geomorphological response to Quaternary climate change e.g.,^8,74^. The position of the Winter-rain-dominated region may have shifted up to 2 degrees northwards during stadials in the Quaternary^75^. In the Summer-rain-dominated areas, ephermal and perennial rivers with headwaters in the Pre-Cordillera or Altiplano, and areas above 2000 m, also respond readily to Quaternary climate change e.g.,^15,22,56,60^.

The evidence for a recent (Pliocene) onset of aridity e.g.,^12,13,18,50,61,70^ is sourced from the same regions that are demonstrably sensitive to Quaternary climate changes (Fig. 6). The Summer-rain dominated areas, below 2000 m, reveal the evidence for an early (Miocene or older) onset. North of 22°S, below 2000 m, surfaces of Miocene age (Fig. 6) are regionally widespread^9,21,24,28^, while they constitute rare relicts south of 22°S^6,76^.

The aridity in the Winter-rain-dominated areas of the Atacama is sensitive to long-term secular changes in the sea-surface temperature of the Pacific^71^; and also to the ENSO phenomenon increased rainfall during El Niño-like conditions^73^, as evidenced by the El Niño-induced extreme rain event in 2015^77^. The strengthening of the Humboldt Current since the Late Miocene and particularly during the Pliocene/Pleistocene transition^71^ (Fig. 6) is a likely cause for the late (Plio-/Pleistocene) aridification observed in the Winter-rain region^50,61,76^. Indicators for past aridity, such as material from preserved stable surfaces, are absent in the Winter-rain-dominated areas because of higher rates of erosion^8,50,61,76^, though under favourable conditions relicts remain^6^.

The aridity in the Summer-rain-dominated areas is due to large-scale tropospheric subsidence^3^ and the rain shadow-effect of the Andes^1^, both of which have existed over the long-term. Due to the distant moisture source^78^, changes in the Summer-rain-dominated areas are less sensitive to changes in the offshore SE Pacific, but governed by global changes in temperature; which are commonly, but not necessarily, synchronous^20,79^. Long-term El Niño-like conditions postulated for the Pliocene^79^ that had profound effects in the Winter-rain region^8,50,61,76^, caused only subtle changes in the core of the Atacama e.g.^24^, this study.

A hyper-arid Atacama Desert core with preserved Miocene surfaces is outlined in Fig. 6 by yellow stippled line. This region is within the present day 2 mm/yr isohyet^1,4^, but would have been smaller due to the northward migration of the winter-rainfall area during glacials^75,80^. Surfaces are generally covered by gypsum soils (Chuca and Costra, sensu Ericksen^37^) and lack signs of recent run-off erosion. Fluvial channels are often dissected by fault activity^24,31^ and in some cases we observed in the field that their bottoms are filled with gypsum dust and/or (un-reworked) volcanic ash.

The region studied here is near the southern fringe of the dry core of the Atacama (Fig. 6). The preservation of Miocene surfaces north of the Adamito fault demonstrate that significant fluvial landscape modification was absent. The headwaters of the paleo-catchments (source area) that supplied the gravels of the fossil alluvial fans north of the Adamito faults are outside the core. Indeed, modern analogues of the channels carrying the gravels show signs of sub-recent fluvial activity in the catchment of the closed basin; the channels are free of the otherwise ubiquitous dust while sediment bars and channels are mostly sharp-edged. The >50 m thick fluvial and lacustrine sediments (cored in October & November 2017) in the clay pan abutting the Adamito fault, are a testament to the continued sedimentation that occurred after the channel and fan abandonment we observe. Without the drainage modifications brought about by faulting, the sedimentary surfaces investigated here could well have been buried. The Early- to Mid-Miocene ages obtained from the fan surfaces indicate that a background of (hyper-) arid conditions (Fig. 6), supported the preservation of sediment surfaces that were shed during intermittent wetter periods^21,28^. The presence of clasts with Oligocene exposure ages (Fig. 6) on the early Miocene fans, indicate that at the time of fan deposition remnants of older (older by ≥9–15 Myr) surfaces existed; indicating that a (hyper-) arid climate might have prevailed already during the Oligocene in this part of the Atacama Desert. Considering the position of our study area on the southern fringe of the dry core, it is likely that the core areas further to the North also had a background (hyper-) aridity since the early Oligocene; similarly, old clasts are found near ~19.5°S^9^, Fig. 6.

Oligocene (hyper-) aridity in the core of the Atacama Desert coincides with the global cooling in the Oligocene^20^ (Fig. 6). Global warming at the end of the Oligocene that reversed at the Miocene/Oligocene boundary^20^ appears to coincide with the main phase of deposition and the termination of the regionally important Azapa sediments, respectively^28^. Wetter periods throughout the Miocene and Pliocene largely coincide with globally warmer periods (Fig. 6).

## Conclusions

The core region of the Atacama, here defined as areas in the Coastal Cordillera and the Central Depression between 19°S and 22°S below 2000 m, was predominantly (hyper-) arid throughout the Miocene, possibly even through much of the Oligocene. The (hyper-) aridity was repeatedly interrupted by slightly wetter phases that were synchronous with the climate-induced changes recorded on the Andean foothills^21,28^. Truncation of drainage systems by moderately active faults in conjunction with the prevailing (hyper-) aridity, was instrumental in preserving the sedimentary surfaces. The ancient (Mio-/Oligocene) onset of (hyper-) arid conditions inferred for the study area (and for the core region to the north of it), contradict the evidence that indicates a Pliocene age for the onset. Considering present day climatic gradients and their causes, and how they have evolved in the past, it is consistent that areas in the Winter-rain-dominated regions south of 22°S or above ~2000 m respond strongly to short-term climatic change whereas the core areas of the Atacama are mostly stagnant. Statements on the aridity of the Atacama and its variability are specific to the areas/catchments investigated and generalizing statements on paleoclimate encompassing the entire geographic entity called ‘Atacama Desert’ that are based on point observations are best avoided.

## Methods

We used geological field observations and a high resolution Digital Elevation Model (DEM) of the study area, based on Pléiades 1B data (s. supplementary), and Aster GDEM data to delineate the drainage catchment (closed basin) and calculate slope maps. Optical satellite imagery (Pléiades 1B Multispectral Image) was additionally used to identify geomorphological landscape features, such as alluvial fan extensions and channels. One volcanic ash layer was dated utilizing U/Pb dating of zircons at the University of Frankfurt. The chronology of the alluvial fan development and the incision of the paleo-drainage is reconstructed using cosmogenic nuclide exposure dating. The sampling strategy and calculation of exposure ages are outlined in the following. Zircon U/Pb isotope analysis was conducted with LA-ICP-MS, for methodological details see Frei and Gerdes^81^. Further details and details pertaining to the DEM-generation and U/Pb dating are provided in the supplementary data set.

Cosmogenic exposure ages were determined for quartz clasts sampled from inactive alluvial fan surfaces during field expeditions in 2013, 2014, and 2015. Quartz clasts were sampled from flat fan surfaces (RL14-01-04, RL15-006, and RL15-007) and from a feeder channel (RL15-005) (Fig. 2A). Furthermore, quartz clasts from a surface isolated by the incision of the paleo-channel were sampled (RL15-001), as well as pebbles in the western paleo channel (RL13-001). The sampling sites on the central and eastern alluvial fans (RL14-01-04, RL15-007) and the isolated surface (RL15-001) are essentially flat, at each location quartz clasts were collected from an area of about 0.5 ha. On the western fan, which had minor relief due to shallow channel incision (see above), the samples were taken exclusively on dispersed interfluves (RL15-006). Pebbles from the feeder channel (RL15-005) and the paleo-channel (RL13-001) were sampled on local highs (former sand bars) in the centre of the channel, remote from material shed from the channel’s hill-slopes.

The majority of the sampled quartz clasts have a reddish-brown desert varnish. They often retain rounded shapes from fluvial transport, occasionally modified by fragments spallation. In instances of ‘kernsprung’, indicated by a localized cluster (usually <2 m diameter) of quartz fragments, only one fragment per cluster was sampled and no other samples collected within a 5 m radius. Dimensions of sampled clasts range between 2–5 cm. The abundance of quartz, of the required clast size (>2 cm), on the surfaces is very low, commonly less than one clast per 25 m^2^. Accordingly attempts at retrieving quartz from the subsurface were not successful. Furthermore, cemented *costra*^37^ in the shallow subsurface, rendered depth profile sampling^48,82^ impossible. Instead, to identify and correct for possible pre-exposure and exhumation, we utilize the multiple-clast approach^83,84^, using 5–7 samples per sampling site.

The individual quartz clasts were crushed, sieved to retain the 250–710 µm grain size; then etched several times in a dilute HF-HNO_3_ mixture^85^. Splits of the etched material were used for ^10^Be and ^21^Ne analysis. ^10^Be sample preparation and measurement followed single stacked column approach detailed in^86^. ^10^Be/^9^Be ratios were measured on CologneAMS^87^, normalized to the ICN standard dilution series values reported by Nishiizumi, *et al*.^47^. ^21^Ne analyses were performed at SUERC following the procedures outlined in^88^.

Exposure ages were calculated using the LSD scaling scheme of Lifton, *et al*.^89^ as implemented in version 3 of ‘the online calculators formerly known as the CRONUS-Earth online calculators’ https://hess.ess.washington.edu/math/v3/v3_age_in.html described in^90^; see also supplement. At the latitude and altitude of the study area, exposure ages calculated with the LSD scaling are 22% lower than those calculated with Stone (based on ‘Lal magnetic’) scaling^89^. We assume a time integrated linear uplift 40 m/Ma^9,91^; since 20 Ma, and report uplift-corrected ages accordingly (see supplement).

## Electronic supplementary material

Supplementary Dataset

## Data Availability

All data generated or analysed during this study are included in this published article (and its Supplementary Information files).

## References

[1] Houston J (2006). Variability of precipitation in the Atacama Desert: its causes and hydrological impact. International Journal of Climatology.

[2] Muñoz, J. Levantamiento Hidrogeológico para el desarrollo de nuevas fuentes de agua en áreas prioritarias de la zona norte de Chile, regiones XV, I, II Y III. Etapa II. (2009).

[3] Takahashi K, Battisti DS (2007). Processes controlling the mean tropical Pacific precipitation pattern. Part II: The SPCZ and the southeast Pacific dry zone. Journal of Climate.

[4] Houston J, Hartley AJ (2003). The Central Andean west-slope rainshadow and its potential contribution to the origin of hyper-aridity in the Atacama desert. International Journal of Climatology.

[5] Houston J (2006). Evaporation in the Atacama Desert: An empirical study of spatio-temporal variations and their causes. Journal of Hydrology.

[6] Nishiizumi K, Caffee MW, Finkel RC, Brimhall G, Mote T (2005). Remnants of a fossil alluvial fan landscape of Miocene age in the Atacama Desert of northern Chile using cosmogenic nuclide exposure age dating. Earth and Planetary Science Letters.

[7] Kober F (2007). Denudation rates and a topography-driven rainfall threshold in northern Chile: Multiple cosmogenic nuclide data and sediment yield budgets. Geomorphology.

[8] Placzek CJ, Matmon A, Granger DE, Quade J, Niedermann S (2010). Evidence for active landscape evolution in the hyperarid Atacama from multiple terrestrial cosmogenic nuclides. Earth and Planetary Science Letters.

[9] Dunai TJ, González López GA, Juez-Larré J (2005). Oligocene–Miocene age of aridity in the Atacama Desert revealed by exposure dating of erosion-sensitive landforms. Geology.

[10] Evenstar LA (2009). Multiphase development of the Atacama Planation Surface recorded by cosmogenic 3He exposure ages: Implications for uplift and Cenozoic climate change in western South America. Geology.

[11] Rech JA, Currie BS, Michalski G, Cowan AM (2006). Neogeneclimate change and uplift in the Atacama Desert, Chile. Geology.

[12] Hartley AJ, Chong G (2002). Late Pliocene age for the Atacama Desert: Implications for the desertification of western South America. Geology.

[13] Sáez A (2012). The stratigraphic record of changing hyperaridity in the Atacama desert over the last 10 Ma. Earth and Planetary Science Letters.

[14] Sillitoe RH, McKee EH (1996). Age of supergene oxidation and enrichment in the Chilean porphyry copper province. Economic Geology.

[15] Latorre C, Betancourt JL, Arroyo MTK (2006). Late Quaternary vegetation and climate history of a perennial river canyon in the Río Salado basin (22°S) of Northern Chile. Quaternary Research.

[16] Rech JA, Quade J, Hart WS (2003). Isotopic evidence for the source of Ca and S in soil gypsum, anhydrite and calcite in the Atacama Desert, Chile. Geochim. Cosmochim. Acta.

[17] Nester PL, Gayo E, Latorre C, Jordan TE, Blanco N (2007). Perennial stream discharge in the hyperarid Atacama Desert of northern Chile during the latest Pleistocene. Proceedings of the National Academy of Sciences of the United States of America.

[18] Hartley AJ, Rice CM (2005). Controls on supergene enrichment of porphyry copper deposits in the Central Andes: A review and discussion. Mineralium Deposita.

[19] Cristini L, Grosfeld K, Butzin M, Lohmann G (2012). Influence of the opening of the Drake Passage on the Cenozoic Antarctic Ice Sheet: a modeling approach. Palaeogeography, Palaeoclimatology, Palaeoecology.

[20] Zachos J, Pagani M, Sloan L, Thomas E, Billups K (2001). Trends, rhythms and abberrations in Global Climate 65 Ma to present. Science.

[21] Jordan, T. E. *et al* Landscape modification in response to repeated onset of hyperarid paleoclimate states since 14 Ma, Atacama Desert, Chile. *Geological Society of America Bulletin*, 10.1130/b30978.1 (2014).

[22] Betancourt JL, Latorre C, Rech JA, Quade J, Rylander KA (2000). A 22,000-year record of Monsoonal precipitation from Northern Chile’s Atacama Desert. Science.

[23] Allmendinger RW, Gonzalez G, Yu J, Hoke G, Isacks B (2005). Trench-parallel shortening in the Northern Chilean Forearc: Tectonic and climatic implications. Geological Society of America Bulletin.

[24] Carrizo D, Gonzalez G, Dunai T (2008). Neogene constriction in the northern Chilean Coastal Cordillera: Neotectonics and surface dating using cosmogenic ^21^Ne. Revista Geologica De Chile.

[25] Reijs J, McClay K (1998). Salar Grande pull-apart basin, Atacama fault system, northern Chile. Geological Society, London, Special Publications.

[26] Coira B, Davidson J, Mpodozis C, Ramos V (1982). Tectonic and magmatic evolution of the Andes of northern Argentina and Chile. Earth-Science Reviews.

[27] Mortimer C (1973). The Cenozoic history of the southern Atacama Desert, Chile. Journal Geol. Soc. Lond..

[28] Evenstar L (2017). Geomorphology on geologic timescales: Evolution of the late Cenozoic Pacific paleosurface in Northern Chile and Southern Peru. Earth-Science Reviews.

[29] McCaffrey R (1996). Estimates of modern arc-parallel strain rates in fore arcs. Geology.

[30] Skarmeta, J. & Marinovic, N. *Hoja Quillagua: región de Antofagasta: carta geológica de Chile escala 1: 250*.*000*. (Instituto de Investigaciones Geológicas, 1981).

[31] Allmendinger RW, González G (2010). Invited review paper: Neogene to Quaternary tectonics of the coastal Cordillera, northern Chile. Tectonophysics.

[32] Denny, C. S. *Alluvial fans in the Death Valley region*, *California and Nevada*. (US Government Printing Office, 1965).

[33] Chong G, Mendoza M, García-Veigas J, Pueyo JJ, Turner P (1999). Evolution and geochemical signatures in a Neogene forearc evaporitic basin: the Salar Grande (Central Andes of Chile). Palaeogeography, Palaeoclimatology, Palaeoecology.

[34] Sáez A, Cabrera L, Jensen A, Chong G (1999). Late Neogene lacustrine record and palaeogeography in the Quillagua–Llamara basin, Central Andean fore-arc (northern Chile). Palaeogeography, Palaeoclimatology, Palaeoecology.

[35] Rodwell MJ, Hoskins BJ (2001). Subtropical anticyclones and summer monsoons. Journal of Climate.

[36] Egger J (2005). Diurnal circulation of the Bolivian Altiplano. Part I: observations. Monthly weather review.

[37] Ericksen, G. E. *Geology and origin of the Chilean nitrate deposits*. Report No. 1188, 37 (USGS, Washington, 1981).

[38] Wang F (2015). Beryllium-10 concentrations in the hyper-arid soils in the Atacama Desert, Chile: Implications for arid soil formation rates and El Niño driven changes in Pliocene precipitation. Geochimica et Cosmochimica Acta.

[39] Michalski G, Bohlke JK, Thiemens M (2004). Long term atmospheric deposition as the source of nitrate and other salts in the Atacama Desert, Chile: New evidence from mass-independent oxygen isotopic compositions. Geochimica et Cosmochimica Acta.

[40] Wells SG, McFadden LD, Poths J, Olinger CT (1995). Cosmogenic 3He surface-exposure dating of stone pavements: Implications for landscape evolution in deserts. Geology.

[41] Parsons, A. J. & Abrahams, A. D. In *Geomorphology of Desert Environments*. (Springer, 2009).

[42] Cereceda P, Larrain H, Osses P, Farías M, Egaña I (2008). The spatial and temporal variability of fog and its relation to fog oases in the Atacama Desert, Chile. Atmospheric Research.

[43] Amidon WH (2016). U-Pb ages of detrital and volcanic zircons of the Toro Negro Formation, northwestern Argentina: Age, provenance and sedimentation rates. Journal of South American Earth Sciences.

[44] Barquero-Molina, M. *40Ar/39Ar chronology and paleomagnetism of ignimbrites and lavas from the central volcanic zone*, *northern Chile*, *and 40Ar/39Ar chronology of silicic ignimbrites from Honduras and Nicaragua*. (University of Wisconsin–Madison, 2003).

[45] Salisbury MJ (2010). 40Ar/39Ar chronostratigraphy of Altiplano-Puna volcanic complex ignimbrites reveals the development of a major magmatic province. Geological Society of America Bulletin.

[46] Kern JM (2016). Geochronological imaging of an episodically constructed subvolcanic batholith: U-Pb in zircon chronochemistry of the Altiplano-Puna Volcanic Complex of the Central Andes. Geosphere.

[47] Nishiizumi K (2007). Absolute calibration of Be-10 AMS standards. Nucl. Instr. Meth. Phys. Res. B.

[48] Dunai, T. J. *Cosmogenic Nuclides: Principles*, *concepts and applications in the Earth surface sciences*. (Cambridge University Press, 2010).

[49] González, G., Dunai, T. J., Carrizo, D. & Allmendinger, R. Young displacements on the Atacama Fault System, northern Chile from field observations and cosmogenic Ne-21 concentrations. *Tectonics* **25**, 10.1029/2005TC001846 (2006).

[50] Jungers MC (2013). Active erosion–deposition cycles in the hyperarid Atacama Desert of Northern Chile. Earth and Planetary Science Letters.

[51] Binnie, A., Binnie, S. A., Parteli, E. J. R. & Dunai, T. J. The implications of sampling approach and geomorphological processes for cosmogenic ^10^Be exposure dating of marine terraces. *Nuclear Instruments and Methods- B: Beam Interactions with Materials and Atoms* (submitted).

[52] Anderson RS, Repka JL, Dick GS (1996). Explicit treatment of inheritance in dating depositional surfaces using *in situ*^10^Be and 26Al. Geology.

[53] Repka JL, Anderson RS, Finkel RC (1997). Cosmogenic dating of fluvial terraces, Fremont River, Utah. Earth and Planetary Science Letters.

[54] Lisiecki, L. E. & Raymo, M. E. A Pliocene‐Pleistocene stack of 57 globally distributed benthic δ18O records. *Paleoceanography* **20** (2005).

[55] Ritter B, Binnie SA, Stuart FM, Wennrich V, Dunai TJ (2018). Evidence for multiple Plio-Pleistocene lake episodes in the hyperarid Atacama Desert. Quaternary Geochronology.

[56] Gayo EM (2012). Late Quaternary hydrological and ecological changes in the hyperarid core of the northern Atacama Desert (~21°S). Earth-Science Reviews.

[57] Rech JA (2010). Evidence for the development of the Andean rain shadow from a Neogene isotopic record in the Atacama Desert, Chile. Earth and Planetary Science Letters.

[58] Kirk-Lawlor N, Jordan TL, Rech JA, Lehman SB (2013). Late Miocene to Early Pliocene paleohydrology and landscape evolution of Northern Chile, 19° to 20°S. Palaegeography Palaeoclimatology Palaeoecology.

[59] Evenstar, L. *et al*. Miocene-Pliocene climate change in the Peru-Chile desert. *6th International symposium on Andean Geodynamics* (ISAG 2005, Barcelona) (2005).

[60] Latorre C (2013). Late Pleistocene human occupation of the hyperarid core in the Atacama Desert, northern Chile. Quaternary Science Reviews.

[61] Amundson R (2012). Geomorphologic evidence for the late Pliocene onset of hyperaridity in the Atacama Desert. Geological Society of America Bulletin.

[62] Maldonado A, Betancourt JL, Latorre C, Villagran C (2005). Pollen analyses from a 50 000-yr rodent midden series in the southern Atacama Desert (25° 30′S). Journal of Quaternary Science.

[63] Quade J (2008). Paleowetlands and regionalclimate change in the central Atacama Desert, northern Chile. Quaternary Research.

[64] Díaz FP, Latorre C, Maldonado A, Quade J, Betancourt JL (2012). Rodent middens reveal episodic, long-distance plant colonizations across the hyperarid Atacama Desert over the last 34,000 years. Journal of Biogeography.

[65] Bozkurt D, Rondanelli R, Garreaud R, Arriagada A (2016). Impact of Warmer Eastern Tropical Pacific SST on the March 2015 Atacama Floods. Monthly Weather Review.

[66] Wilcox AC (2016). An integrated analysis of the March 2015 Atacama floods. Geophysical Research Letters.

[67] Hartley AJ, Evenstar L (2010). Cenozoic stratigraphic development in the north Chilean forearc: Implications for basin development and uplift history of the Central Andean margin. Tectonophysics.

[68] Knighton, A. & Nanson, G. Distinctiveness, diversity and uniqueness in arid zone river systems. *Arid Zone Geomorphology: Process, Form and Change in Drylands*, Thomas, D. S. G. (ed.). 2nd Edition (John Wiley & Sons, 185–203, 1997).

[69] Mortimer C, Saric N (1972). Landform evolution in the coastal region of Tarapacá Province, Chile. Revue de géomorphologie dynamique.

[70] Oerter E (2016). Early to middle Miocene climate in the Atacama Desert of northern Chile. Palaeogeography, Palaeoclimatology, Palaeoecology.

[71] Garreaud RD, Molina A, Farias M (2010). Andean uplift, ocean cooling and Atacama hyperaridity: A climate modeling perspective. Earth and Planetary Science Letters.

[72] Schlunegger, F., Norton, K. P., Delunel, R., Ehlers, T. A. & Madella, A. Late Miocene increase in precipitation in the Western Cordillera of the Andes between 18–19°S latitudes inferred from shifts in sedimentation patterns. *Earth and Planetary Science Letters* (2017).

[73] Garreaud RD, Vuille M, Compagnucci R, Marengo J (2009). Present-day South American climate. Palaeogeography Palaeoclimatology Palaeoecology.

[74] De Porras M, Maldonado A, Pol‐Holz D, Latorre C, Betancourt J (2017). Late Quaternary environmental dynamics in the Atacama Desert reconstructed from rodent midden pollen records. Journal of Quaternary Science.

[75] Lamy F, Klump J, Hebbeln D, Wefer G (2000). Late Quaternary rapidclimate change in northern Chile. Terra Nova.

[76] Placzek C, Granger DE, Matmon A, Quade J, Ryb U (2014). Geomorphic process rates in the central Atacama Desert, Chile: Insights from cosmogenic nuclides and implications for the onset of hyperaridity. American Journal of Science.

[77] Scott C, Lohman R, Jordan T (2017). InSAR constraints on soil moisture evolution after the March 2015 extreme precipitation event in Chile. Scientific Reports.

[78] Ehlers TA, Poulsen CJ (2009). Influence of Andean uplift on climate and paleoaltimetry estimates. Earth and Planetary Science Letters.

[79] Fedorov A (2006). The Pliocene paradox (mechanisms for a permanent El Niño). Science.

[80] Stuut JBW, Lamy F (2004). Climate variability at the southern boundaries of the Namib (Southwestern Africa) and Atacama (northern Chile) coastal deserts during the last 120,000 yr. Quaternary Research.

[81] Frei D, Gerdes A (2009). Precise and accurate *in situ* U–Pb dating of zircon with high sample throughput by automated LA-SF-ICP-MS. Chemical Geology.

[82] Hein AS (2009). Middle Pleistocene glaciation in Patagonia dated by cosmogenic-nuclide measurements on outwash gravels. Earth and Planetary Science Letters.

[83] Binnie A (2016). Accelerated late quaternary uplift revealed by 10 Be exposure dating of marine terraces, Mejillones Peninsula, northern Chile. Quaternary Geochronology.

[84] Farbod Y (2016). Spatial variations in late Quaternary slip rates along the Doruneh Fault System (Central Iran). Tectonics.

[85] Kohl CP, Nishiizumi K (1992). Chemical isolation of quartz for measurement of *in-situ* produced cosmogenic nuclides. Geochim. Cosmochim. Acta.

[86] Binnie, S. A. *et al*. Separation of Be and Al for AMS using single-step column chromatography. *Nuclear Instruments and Methods in Physics Research Section B: Beam Interactions with Materials and Atoms* (2015).

[87] Dewald A (2013). CologneAMS, a dedicated center for accelerator mass spectrometry in Germany. Nuclear Instruments & Methods in Physics Research Section B-Beam Interactions with Materials and Atoms.

[88] Codilean AT (2008). Single-grain cosmogenic ^21^Ne concentrations in fluvial sediments reveal spatially variable erosion rates. Geology.

[89] Lifton N, Sato T, Dunai TJ (2014). Scaling *in situ* cosmogenic nuclide production rates using analytical approximations to atmospheric cosmic-ray fluxes. Earth and Planetary Science Letters.

[90] Balco G, Stone JO, Lifton NA, Dunai TJ (2008). A complete and easily accessible means of calculating surface exposure ages or erosion rates from (10)Be and (26)Al measurements. Quaternary Geochronology.

[91] Evenstar LA, Stuart FM, Hartley AJ, Tattitch B (2015). Slow Cenozoic uplift of the western Andean Cordillera indicated by cosmogenic ^3^He in alluvial boulders from the Pacific Planation Surface. Geophysical Research Letters.

[92] Quezada, A., Vasquez, P., Sepúlveda, F., Blanco, N. & Tomlinson, A. J. Mapa Compilación Geológica Área Quillagua - Salar Grande 1:100.000. *Servicio Nacional de Geologica y Mineria Gobierno Regional de Tarapacá* (2012).

[93] Alpers CN, Brimhall GH (1988). Middle Miocene climatic change in the Atacama Desert, northern Chile: Evidence from supergene mineralization at La Escondia. Geol. Soc. Am. Bull..

[94] Arancibia G, Matthews S (2006). & de Arce, C. P. K–Ar and 40Ar/39Ar Geochronology of supergene processes in the Atacama Desert, northern Chile: Tectonic and climatic relations. Journal of the Geological Society.

[95] Bouzari F, Clark AH (2002). Anatomy, evolution, and metallogenic significance of the supergene orebody of teh Cerro Colorado porpyry copper deposit, I region, northern Chile. Economic Geology.

[96] Sun, T., Bao, H., Reich, M. & Hemming, S. R. More than Ten Million Years of Hyper-aridity recorded in the Atacama Gravels. *Geochimica et Cosmochimica Acta* (2018).

[97] Sáez A, Godfrey LV, Herrera C, Chong G, Pueyo JJ (2016). Timing of wet episodes in Atacama Desert over the last 15 ka. The Groundwater Discharge Deposits (GWD) from Domeyko Range at 25°S. Quaternary Science Reviews.

[98] Owen, J. *et al*. In *AGU Fall Meeting*. Abstract T31C- 0857 (AGU) (2003).

[99] Clark AH, Tosdal RM, Ferrar E, Plazolles A (1990). Geomorphologic envrionment and age of supergene enrichment of the Cuajone, Quellaveco, and Toquepala porphyry copper deposits, southeastern Peru. Economic Geology.

