# Supplementary material for: Neogene fluvial landscape evolution in the hyperarid core of the Atacama Desert
Source: Sci Rep. 2018 Sep 17;8:13952. doi: 10.1038/s41598-018-32339-9 (PMC6141514; doi:10.1038/s41598-018-32339-9)
Supplement: Supplementary file 1 — Supplementary Dataset [file 41598_2018_32339_MOESM1_ESM.pdf]

Supplementary data

"Neogene fluvial landscape evolution in the hyperarid core of the Atacama Desert"

Benedikt Ritter[a*], Finlay M. Stuart[b], Steven A. Binnie[a], Axel Gerdes[c], Volker Wennrich[a], Tibor J. Dunai[a]

[a] Institute of Geology & Mineralogy, University of Cologne, Germany

[b] Isotope Geosciences Unit, Scottish Universities Environmental Research Centre, East Kilbride, UK

[c] Institute of Geosciences, Goethe-University Frankfurt, Germany

Methods

Digital Elevation Model (DEM) Extraction

The Digital Elevation Model (DEM) was generated using a high resolution stereo satellite image set obtained from Pléiades 1B system. Image recording was on 05.05.2015 with a ground resolution of 0.5 m in the panchromatic band. ExelisVIS ENVI 5.1 was used to derive a surface model with the DEM Extraction module. Tie point determination between the two stereo images was conducted manually. Post-processing tools, including filter methods to reduce spikes (ENVI DEM Editing tools), were applied. The final model has a resolution of 1 m. A pansharped image was produced using ExelisVIS ENVI 5.1. Optical satellite imagery (Pléiades 1B Multispectral Image) was used to identify landscape features, such as alluvial fan extensions and channels. Further GIS analysis was obtained by using ArcGIS® 10.5.1 (ESRI http://www.esri.com), such as watershed delineation or hill shading (Spatial Analyst – Hydrology). Swath profiles were calculated using the high resolution Pléiades 1B DEM and focal statistics (Spatial Analyst ArcGIS® 10.5.1). Slope map (Fig. 6C in supplementary) was calculated using ArcGIS 10.5.1 using Spatial Analyst – Slope tool on an aggregated DEM (5x5 m).

Tephra U/Pb dating

Tephra sample T4 was processed using standard mineral separation techniques at the University of Cologne. Isotope mass spectrometry was conducted at the Institute for Geosciences at Frankfurt University. Hand-picked zircon grains were mounted in a 25 mm diameter circular epoxy mount and polished to expose their grain interiors. The internal structure of zircons was investigated using cathodoluminescence imaging. Zircon U/Pb isotope analysis was conducted with laser ablation inductively coupled plasma mass spectrometry (LA-ICP-MS), for methodological details see Frei and Gerdes [1]. The laser-ICPMS U–Pb data of the detrital zircons (n= 62) are reported with 2σ uncertainties in Table 3.

Cosmogenic nuclide exposure dating

Samples were ground and sieved to 250-1000 µm and subsequently purified by sequential HF-leaching [2]. Purified quartz separates were investigated under a microscope. Those with a high abundance of visible fluid inclusions and/or fragments resembling chalcedony were further etched or excluded for ^{21}Ne. Inductively coupled plasma optical emission spectrometry (ICP-OES) was used to verify the purity of the quartz before dissolution.

^{10}Be target for accelerator mass spectrometry (AMS) were prepared using the stacked column approach outlined in Binnie et al. [3]. A reagent blank was prepared in tandem with the sample batch ^{10}Be/^{9}Be AMS measurements were made on CologneAMS [4] normalized to the ICN standard dilutions prepared by Nishiizumi [5]. Concentrations of ^{10}Be are reported following blank subtraction, which was less than 1% of the total number of nuclides measured in each case. The 1 standard deviation analytical precision of the nuclide concentrations was estimated by summing in quadrature the relative uncertainties on the AMS measurements of both the samples and the blank, along with a 1% (1 s.d.) estimate for the precision of the mass of ^{9}Be added during spiking.

For cosmogenic ^{21}Ne, cleaned and purified samples were sieved to <125 µm and packed into aluminium foil cups. The samples were measured with a noble-gas mass spectrometer at Scottish Universities Environmental Research Centre (SUERC) applying the standard procedure [6-8].

For age calculation, we used a ^{10}Be half-life of 1.36 ± 0.07 Ma [5] and a 'nuclide dependent scaling' after Lifton, et al. [9], calculated with "The online exposure age calculator formerly known as the CRONUS-Earth online exposure age calculator." Version 3, http://hess.ess.washington.edu/math/v3/v3_age_in.html; [10]. This employed a high-latitude, sea-level spallogenic ^{10}Be production rate of 3.92 ± 0.31 atoms g^{-1} a^{-1} [11] and for ^{21}Ne 18.7 ± 2.3 atoms $g^{-1}a^{-1}$ [12] and ERA40 atmospheric model.

We applied a time-integrated inverse modelling of cosmogenic nuclide production rates by subsequent modelled subsidence to paleo elevations. We used a linear uplift model of 40 m/Ma [13,14] with uplift commencing at 20 Ma (assuming that uplift of the alluvial fans started from a vertical position near sea-level). To apply uplift correction using LSDn scaling as implemented the CRONUS-Earth online calculator (V.3), we calculated the uplift-dependant change by using an erosion rate (the reduction of the mass air above the sample by uplift is mathematically equivalent to erosion).

The density of air decreases with altitude (rising from sea-level to 1100 m, our mean sample elevation) the density of air is reduced by~10% (U.S. Standard Atmosphere 1976, NASA-TM-X-74335). For the purpose of calculating the effect of atmospheric mass removal by uplift we utilize the mean density of air at 500 m.a.s.l. (1.167 kg/m^3, NASA-TM-X-74335). The $\rho_{air\ 500m}/\rho_{Qtz}$ multiplied by the uplift rate provides the 'erosion rate' (in our case 1,762 x*10^{-6} cm/yr) for input into the online calculator. Since we use the density of air at 500 m at all elevations, we assign a 5% internal uncertainty for our uplift correction (caused by the utilization of a mean density of air).

For samples with concentrations implying an age >20 Ma, we use the scaling calculated for a virtual 20 Ma sample at the sampling location, to calculate the exposure prior to 20 Ma (from the concentration that is in excess of that produced since 20 Ma); which is added to 20 Ma to obtain the exposure age.

The uplift correction is relatively insensitive to deviation of the assumption of linear uplift (within the bounds of the same long-term average). Ages change by ±10% if the uplift rate decreased/increased fourfold after half the exposure time (Dunai et al. 2005). We assign a ±10% external uncertainty to the uplift-corrected ages. Ages without uplift correction would be younger. Early Miocene ages would be younger by ~30%, differences decrease towards younger ages (~2% at 2 Ma).

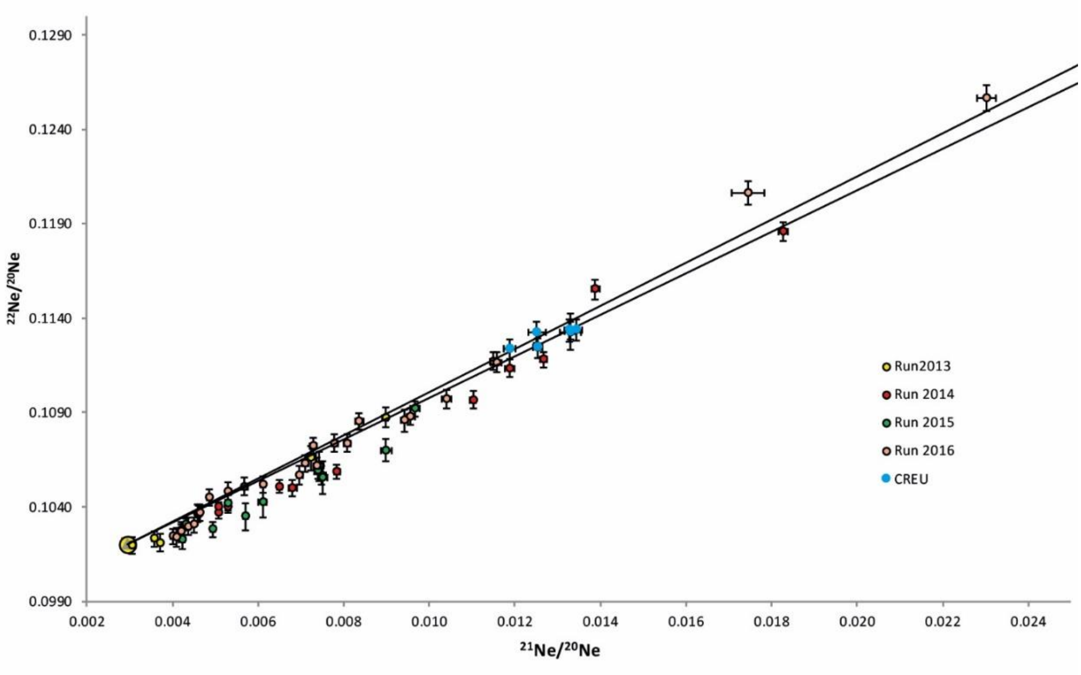

Fig. 2 Neon isotope data of all analysis reported in this paper. The lines represent a mixture of air and the spallation-derived Ne in quartz. Sample and standard (CREU, Vermeesch et al., 2015) measurements are shown.

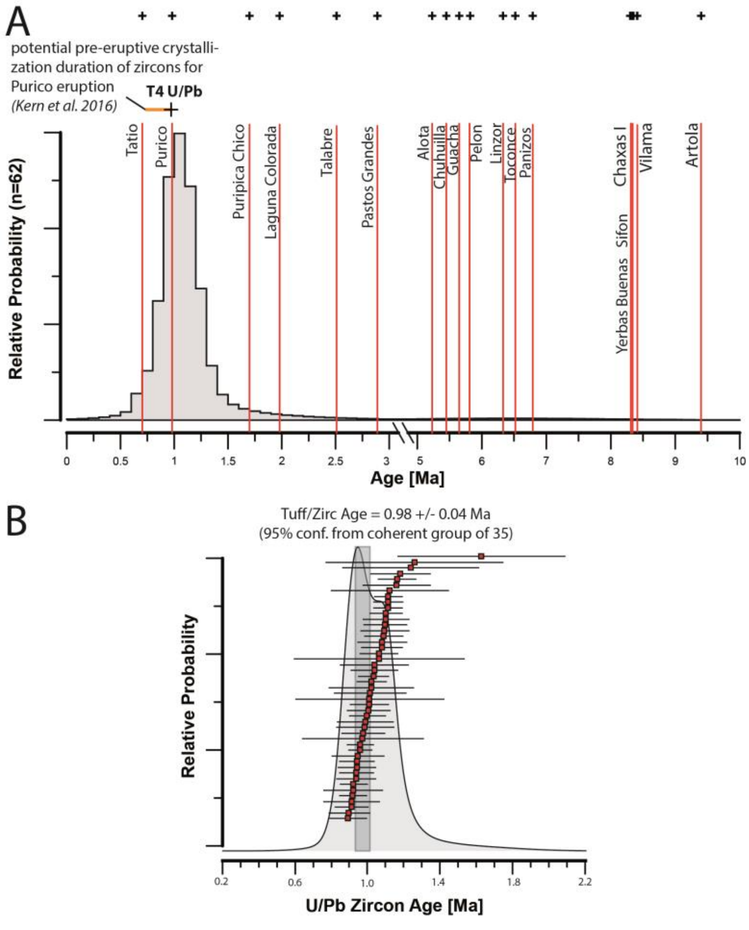

Fig. 3 (A) The zircon U/Pb age record is represented by histograms and associated probability density function curve for zircons ages between 0 and 10Ma, determined for the sampled tephra T4. Note that the scale of age axis is interrupted and changes between 3 and 5 Ma. Eruption ages (Ar/Ar) of adjacent volcanic complexes are marked as vertical bars [15]. Orange bar indicates potential pre-eruptive crystallization duration of zircons within the Purico magma chamber [16]. U/Pb zircon ages could therefore be related to the Purico or the Tatio eruptions. (B) Rank order plots (ROP) and zircon U/Pb ages as probability density function (PDF) curves. The shaded PDF curve represents the distribution for the ROP displayed. The normal distribution is interpreted to represent a single population of crystals from an individual eruption. Horizontal lines display 2σ analytical uncertainty of data from which cumulative probability curves were calculated. The age of 0.98 ± 0.04 is the weighted mean and 2σ error for the dominant peak in the distribution of 35 zircons of T4.

Additional Site Data

The easternmost fan (Fan East) has a maximum width of 1.5 km and length of 4 km, moderately dipping (1.46°) to the north. The lower 1.5 km are buried under lacustrine sediments, related to lake stages in the Quillagua-Llamara basin [17]. Diatom-rich sediments indicate deposits of the Quillagua Formation 5.5-4.5 Ma, [17,18] outcrop at the surface. Deflation removed material to the bed-rock level adjacent to the fans resulting that the fan surface is elevated approximately 5-10 m above the adjacent surface, resembling an inverted landscape topography in the lower part of the fan.

The central fan (Fan Center) has a maximum length of 3 km, including the final 1 km covered by lacustrine sediments, and a maximum width of 2.4 km. Similar like the eastern fan, the center fan is surrounded by an inverted landscape-like topography. The fan surface dips moderately to the north (1.29°). A feeder channel (RL15-005) is well preserved and has incised into blocks of the Adamito Fault System, indicating synchronous evolution/activity.

The determination of the outline of the western-most fan (Fan West) is difficult; is due to significant surface modification by fluvial erosion in the distal portions, and dust cover approaching its apex. To the west, the fan is bound by the Atacama Fault. The east is limited by deflation sinks, and the western margin of the Central Fan in its lower section. The surface dips moderately to the north (0.93°), with a maximum width of 2 km and maximum length of 4.5 km. Fan sediments are buried under lacustrine sediments in the distal part.

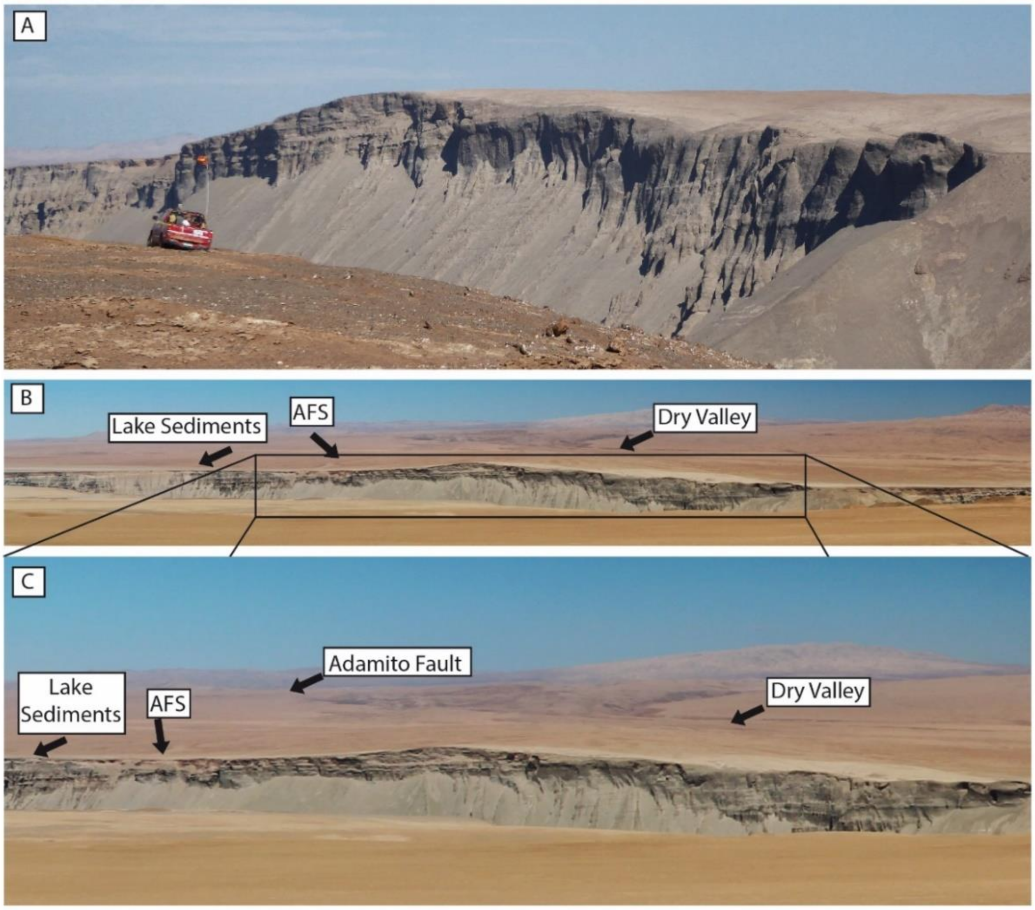

Fig. 4 Photographs (made by B. Ritter) taken from the northern rim of the Rìo Loa canyon towards the transpressional topographic high. Lacustrine sediments are exclusively found to the east of this high (left side in the images). Fluvial sediments of a 'Proto-Rio Loa' system are visible on both sides of the AFS.

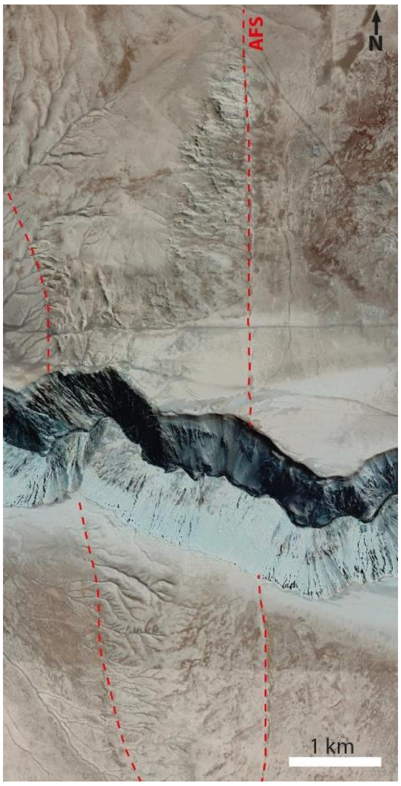

Fig. 5 Google Earth image (Image data: ©2018 CNES/Airbus & Digital Globe, image recoding 11/7/2014) highlighting the course of the splay fault and uplifted tectonic block. Note, the vertical displacement north of the Río Loa.

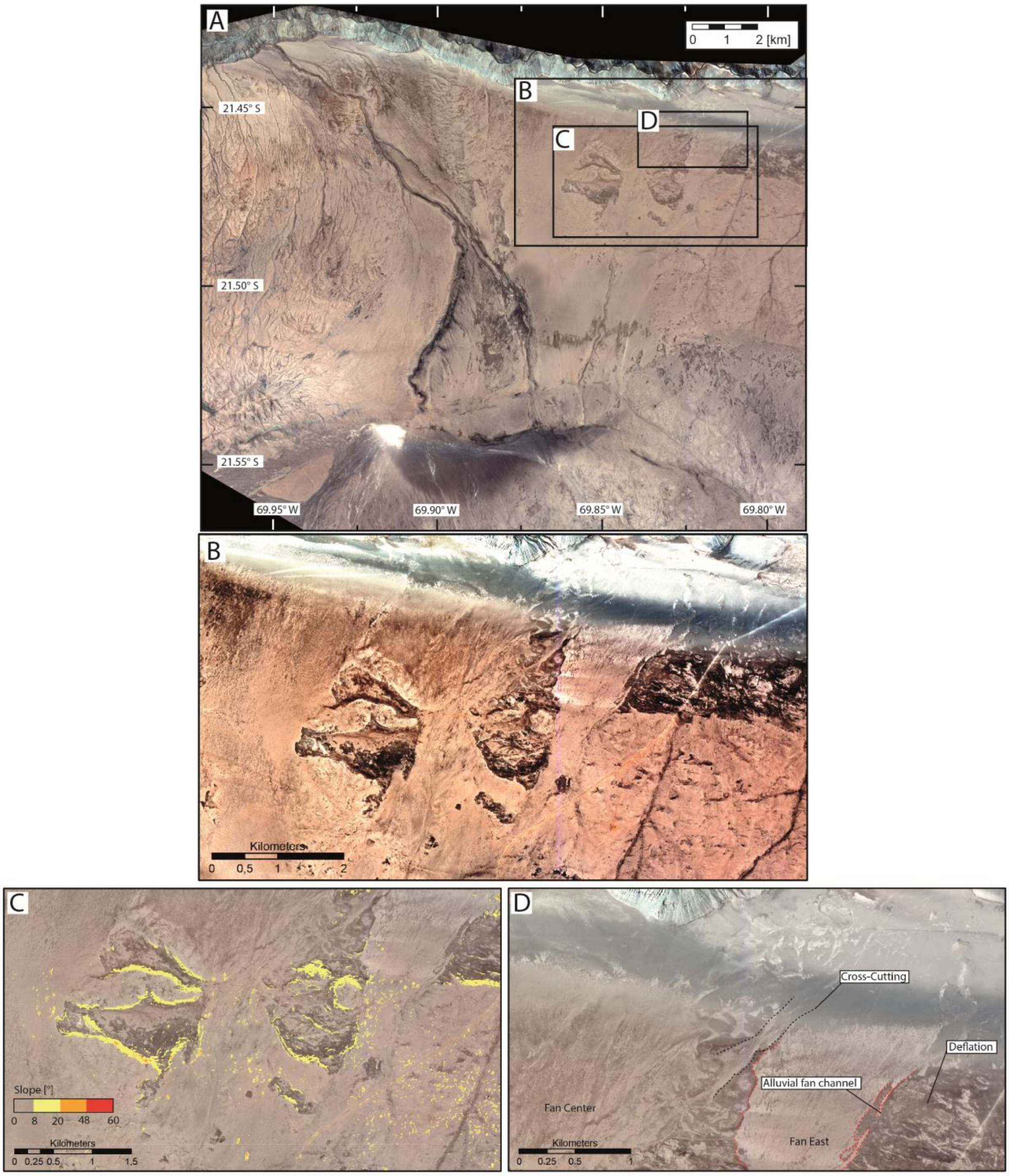

Fig. 6: A) Pléiades 1B Multi-Spectral Image of the study area (maps created using ArcGIS® 10.5.1, ESRI http://www.esri.com). This is identical to Figure 2A. Black rectangles indicate clipping of Fig. 6B-D. B) Enlarge image of depositional fan surfaces north of the Adamito fault. To increase the visibility of boundaries between alluvial fans and inverted landscape, the Pléiades 1B Multi-Spectral Image was modified using contrast enhancement. C) Pléiades 1B Multi-Spectral Image including calculated slope map using ArcGIS 10.5.1® Slope angles >8° are displayed in colour and indicate boundaries between alluvial fan sediments and deflation hollows. D) Enlarge image of the eastern alluvial fan surface north of the Adamito fault (Pléiades 1B Multi-Spectral Image). Visible are former topographic lows (channels with gravel infill), which are now topographic highs due landscape inversion at the eastern limit of the eastern fan. Cross-cutting on the center fan towards the eastern fan is marked.

Table 1: Cosmogenic ^{21}Ne isotope data for quartz clasts. Internal uncertainty 5% of exposure age, external uncertainty 10% of exposure age for samples older than 20 Ma.

			Elevation	Mean Thickness	^{21}Ne	^{21}Ne	Exposure	Internal	External
**Sample**	**Lat.**	**Long.**	**[m]**	**[cm]**	**[atoms/g]**	**Uncert.**	**Age [Ma]**	**Uncert.[Ma]**	**Uncert. [Ma]**
**RL 14/01a**	S 21°28’02.5’’	W 69°49’15.0’’	898	1	5.32E+08	1.6E+07	34.15	1.71	3.42
**RL 14/01b**	S 21°28’02.5’’	W 69°49’15.0’’	898	1.5	3.10E+08	1.0E+07	17.94	0.76	2.01
**RL 14/01c**	S 21°28’02.5’’	W 69°49’15.0’’	898	2	2.90E+08	9E+06	16.55	0.66	1.81
**RL 14/01d**	S 21°28’02.5’’	W 69°49’15.0’’	898	2.5	2.87E+08	9E+06	16.64	0.67	1.82
**RL 14/01e**	S 21°28’02.5’’	W 69°49’15.0’’	898	3.5	2.43E+08	8E+06	13.66	0.53	1.43
**RL 14/01f**	S 21°28’02.5’’	W 69°49’15.0’’	898	3	1.62E+08	5E+06	7.98	0.30	0.77
**RL 14/02**	S 21°28’14.5’’	W 69°50’21.6’’	907	1.5	2.16E+08	8E+06	10.85	0.50	1.12
**RL 14/02b**	S 21°28’14.5’’	W 69°50’21.6’’	907	1.5	3.34E+08	1.1E+07	19.80	0.92	2.31
**RL 14/02c**	S 21°28’14.5’’	W 69°50’21.6’’	907	1	1.37E+08	5E+06	6.14	0.26	0.59
**RL 14/02d**	S 21°28’14.5’’	W 69°50’21.6’’	907	2	1.92E+08	7E+06	9.49	0.37	0.94
**RL 14/02e**	S 21°28’14.5’’	W 69°50’21.6’’	907	1.5	1.70E+08	6E+06	8.06	0.31	0.78
**RL 14/02f**	S 21°28’14.5’’	W 69°50’21.6’’	907	2.5	3.56E+08	1.2E+07	21.41	1.07	2.14
**RL 14/03a**	S 21°28'21.33"	W 69°50'33.8"	910	1.5	1.30E+08	4E+06	5.82	0.21	0.54
**RL 14/03b**	S 21°28'21.33"	W 69°50'33.8"	910	1.5	2.33E+08	1.0E+07	11.94	0.64	1.30
**RL 14/03c**	S 21°28'21.33"	W 69°50'33.8"	910	1.5	1.78E+08	7E+06	8.49	0.35	0.84
**RL 14/03d**	S 21°28'21.33"	W 69°50'33.8"	910	2	2.23E+08	9E+06	11.46	0.52	1.19
**RL 14/03e**	S 21°28'21.33"	W 69°50'33.8"	910	2.25	6.90E+07	2.8E+06	2.88	0.12	0.27
**RL 14/03f**	S 21°28'21.33"	W 69°50'33.8"	910	4	3.28E+08	1.1E+07	21.00	0.99	2.49
**RL 14/04a**	S 21°27’54.1’’	W 69°51’54.4’’	880	1.5	1.25E+08	5E+06	5.71	0.24	0.55
**RL 14/04b**	S 21°27’54.1’’	W 69°51’54.4’’	880	1.25	7.77E+07	3.2E+06	3.28	0.14	0.31
**RL 14/04c**	S 21°27’54.1’’	W 69°51’54.4’’	880	1.75	1.25E+08	4E+06	5.73	0.22	0.54
**RL 14/04d**	S 21°27’54.1’’	W 69°51’54.4’’	880	2.25	1.40E+08	5E+06	6.64	0.29	0.65
**RL 14/04e**	S 21°27’54.1’’	W 69°51’54.4’’	880	2.25	7.38E+07	4.9E+06	3.19	0.22	0.34
**RL 14/04f**	S 21°27’54.1’’	W 69°51’54.4’’	880	2.75	2.00E+08	7E+06	10.46	0.45	1.07
**RL13001a**	S 21°27'35.2’’	W 69°55'07.8’’	764	1	4.05E+07	1.3E+06	1.78	0.06	0.16
**RL13001b**	S 21°27'35.2’’	W 69°55'07.8’’	764	1	1.47E+08	5E+06	7.58	0.28	0.73
**RL13001c**	S 21°27'35.2’’	W 69°55'07.8’’	764	1	1.53E+08	5E+06	7.98	0.29	0.77
**RL13001d**	S 21°27'35.2’’	W 69°55'07.8’’	764	1	6.51E+07	2.3E+06	2.96	0.11	0.27
**RL13001e**	S 21°27'35.2’’	W 69°55'07.8’’	764	1	5.17E+07	2.1E+06	2.28	0.10	0.21
**RL 15-001a**	S 21°28'54.0’’	W 69°53'34.1’’	877	1.75	1.79E+08	9E+06	8.85	0.50	0.94
**RL 15-001b**	S 21°28'54.0’’	W 69°53'34.1’’	877	2.5	2.46E+08	8E+06	13.67	0.58	1.45
**RL 15-001c**	S 21°28'54.0’’	W 69°53'34.1’’	877	2.5	1.81E+08	7E+06	9.20	0.42	0.93
**RL 15-001d**	S 21°28'54.0’’	W 69°53'34.1’’	877	3.5	3.69E+08	1.2E+07	22.34	1.12	2.23
**RL 15-001e**	S 21°28'54.0’’	W 69°53'34.1’’	877	3	2.95E+08	1.0E+07	17.93	0.81	2.03
**RL 15-001f**	S 21°28'54.0’’	W 69°53'34.1’’	877	2.5	2.89E+08	9E+06	17.11	0.72	1.90
**RL 15-005a**	S 21°30'17.3’’	W 69°50'55.8’’	1003	0.75	2.77E+08	9E+06	13.41	0.55	1.41
**RL 15-005b**	S 21°30'17.3’’	W 69°50'55.8’’	1003	0.75	4.18E+08	1.4E+07	25.90	1.29	2.59
**RL 15-005c**	S 21°30'17.3’’	W 69°50'55.8’’	1003	1	2.20E+08	8E+06	9.96	0.40	0.99
**RL 15-005d**	S 21°30'17.3’’	W 69°50'55.8’’	1003	0.75	2.87E+08	1E+07	14.02	0.60	1.49

**RL 15-005e**	S 21°30'17.3''	W 69°50'55.8''	1003	1.5	4.06E+08	1.4E+07	25.01	1.25	2.50
**RL 15-005f**	S 21°30'17.3''	W 69°50'55.8''	1003	1.5	1.18E+08	5E+06	4.77	0.23	0.46
**RL 15-006a**	S 21°28'39.9''	W 69°49'33.8''	930	2	1.61E+08	6E+06	7.46	0.33	0.74
**RL 15-006b**	S 21°28'39.9''	W 69°49'33.8''	930	2.5	2.58E+08	9E+06	13.85	0.58	1.47
**RL 15-006c**	S 21°28'39.9''	W 69°49'33.8''	930	2.5	2.10E+08	7E+06	10.54	0.40	1.06
**RL 15-006d**	S 21°28'39.9''	W 69°49'33.8''	930	2.5	1.07E+08	5E+06	4.69	0.25	0.47
**RL 15-006e**	S 21°28'39.9''	W 69°49'33.8''	930	2.5	1.61E+08	8E+06	7.58	0.40	0.78
**RL 15-006f**	S 21°28'39.9''	W 69°49'33.8''	930	2.5	1.32E+08	6E+06	5.99	0.31	0.60
**RL 15-007a**	S 21°30'14.8''	W 69°50'39.7''	1008	1.5	3.53E+08	1.2E+07	19.21	0.87	2.21
**RL 15-007b**	S 21°30'14.8''	W 69°50'39.7''	1008	2.25	2.31E+08	9E+06	10.91	0.51	1.14
**RL 15-007c**	S 21°30'14.8''	W 69°50'39.7''	1008	2	2.82E+08	9E+06	14.15	0.59	1.50
**RL 15-007d**	S 21°30'14.8''	W 69°50'39.7''	1008	2.5	1.81E+08	7E+06	8.12	0.36	0.81
**RL 15-007e**	S 21°30'14.8''	W 69°50'39.7''	1008	2.5	2.70E+08	1.1E+07	13.55	0.68	1.48
**RL 15-007f**	S 21°30'14.8''	W 69°50'39.7''	1008	3.25	4.46E+08	1.5E+07	27.93	1.40	2.79
**RL 15-007g**	S 21°30'14.8''	W 69°50'39.7''	1008	3.5	2.51E+08	1.0E+07	12.69	0.64	1.37

Table 2: Cosmogenic ^{10}Be isotope data for quartz clasts.

			**Elevation**	**^{10}Be**	**^{10}Be**	**Exposure**	**Internal**	**External**
**Sample**	**Lat.**	**Long.**	**[m]**	**[atoms/g]**	**Uncert.**	**Age [Ma]**	**Uncert.[Ma]**	**Uncert. [Ma]**
**RL 14/01 b**	S 21°28'02.5''	W 69°49'15.0''	898	9.50E+06	3.1E+05	2.89	0.22	0.46
**RL 14/01 c**	S 21°28'02.5''	W 69°49'15.0''	898	1.20E+07	4E+05	saturated	saturated	saturated
**RL 14/01 d**	S 21°28'02.5''	W 69°49'15.0''	898	1.00E+07	3E+05	4.62	0.70	1.44
**RL 14/01 e**	S 21°28'02.5''	W 69°49'15.0''	898	9.87E+06	3.3E+05	5.39	1.15	2.36
**RL 14/02 d**	S 21°28'14.5''	W 69°50'21.6''	907	8.87E+06	2.9E+05	3.09	0.28	0.57
**RL 14/02 e**	S 21°28'14.5''	W 69°50'21.6''	907	7.15E+06	2.4E+05	1.75	0.10	0.21

Table 3: U/Pb analysis data to measured zircons of T4.

LA-ICP-MS data of zircons from T4. Spot size = 30 µm depth of crater ~15 µm. ^{206}Pb/^{238}U error is the quadratic additions of the within run precision (2 SE) and the external reproducibility (2 SD) of the reference zircon. ^{207}Pb/^{206}Pb error propagation (^{207}Pb signal dependent) following Gerdes and Zeh [19]. ^{207}Pb/^{235}U error is the quadratic addition of the ^{207}Pb/^{206}Pb and ^{206}Pb/^{238}U uncertainty.

Grain	^{207}Pba (cps)	Ub (ppm)	Pbb (ppm)	Thb U	^{206}Pbcc (%)	^{206}Pbd ^{238}U	±2s (%)	^{207}Pbd ^{235}U	±2s (%)	^{207}Pbd ^{206}Pb	±2s (%)	rhoe	^{206}Pb ^{238}U	±2s (Ma)	^{207}Pb ^{235}U	±2s (Ma)
A339	114	807	0	1.49	24.4	0.00009955	9.5	0.001588	73	0.1157	73	0.13	0.6	0.1	1.6	1.2
A340	74	355	0	1.45	14	0.0001461	11.2	0.0009503	14	0.0472	8	0.81	0.9	0.1	1	0.1
A341	280	559	0	1.31	31	0.0001387	11.6	0.0009034	15	0.0473	8.7	0.8	0.9	0.1	0.9	0.1
A342	77	624	0	1.39	7.7	0.0001433	8.4	0.0009054	11	0.0458	6.4	0.79	0.9	0.1	0.9	0.1
A343	628	979	0	1.22	35.4	0.0001418	17.2	0.0009271	19	0.0474	8.6	0.89	0.9	0.2	0.9	0.2
A344	1918	1226	0	1.84	60.2	0.0001356	13.6	0.0008498	16	0.0455	8.1	0.86	0.9	0.1	0.9	0.1
A345	1514	350	0	1.43	71.8	0.0001512	34.5	0.0009046	60	0.0434	49	0.57	1	0.3	0.9	0.6
A346	109	753	0	1.75	9.6	0.0001367	8.4	0.0008633	10	0.0458	6.3	0.8	0.9	0.1	0.9	0.1
A347	1760	1367	0	2.4	52.1	0.0001585	23.2	0.001074	25	0.0492	10	0.91	1	0.2	1.1	0.3
A348	6043	964	0	1.68	73.2	0.0001571	40.7	0.001731	97	0.08	88	0.42	1	0.4	1.8	1.7
A349	78	756	0	1.88	6.9	0.000147	15.6	0.0009159	17	0.0452	6.8	0.92	0.9	0.1	0.9	0.2
A351	606	873	0	1.35	49.7	0.0001576	19.8	0.0009938	22	0.0458	8.7	0.91	1	0.2	1	0.2
A352	2073	854	0	2.01	63.4	0.0001803	16.2	0.001137	18	0.0457	8.5	0.88	1.2	0.2	1.2	0.2
A353	1131	649	0	1.4	57.6	0.000161	12.5	0.001009	15	0.0455	8.1	0.84	1	0.1	1	0.2
A354	1314	1090	0	2.26	45.3	0.0001731	7.5	0.001084	12	0.0454	9.4	0.62	1.1	0.1	1.1	0.1
A355	3092	599	0	1.37	54	0.0001923	30.4	0.001788	310	0.0674	310	0.1	1.2	0.4	1.8	5.6
A356	102	713	0	1.34	12.2	0.0001313	12	0.0008292	14	0.0458	8	0.83	0.8	0.1	0.8	0.1
A357	2345	1441	0	2.89	43	0.0001568	10.9	0.001016	13	0.047	7.3	0.83	1	0.1	1	0.1
A358	6096	884	0	1.4	83.1	0.000253	28.4	0.002899	87	0.0831	82	0.33	1.6	0.5	2.9	2.6
A359	8847	497	0	1.26	68.3	0.0009604	18.1	0.006326	20	0.0478	8.2	0.91	6.2	1.1	6.4	1.3
A360	883	554	2	1.05	3.3	0.003094	2.4	0.02016	11	0.0473	11	0.21	19.9	0.5	20.3	2.3
A361	221	898	0	2.13	14.3	0.0001589	8.1	0.001018	10	0.0465	6.4	0.79	1	0.1	1	0.1
A362	4218	722	0	0.9	35.1	0.0001741	29	0.001787	65	0.0745	59	0.44	1.1	0.3	1.8	1.2
A363	2479	1073	0	2.87	45.8	0.0001529	16.3	0.0009696	18	0.046	7.1	0.92	1	0.2	1	0.2
A364	39702	1440	100	0.82	0.2	0.07023	1.6	0.5351	2	0.0553	1.4	0.77	438	7	435	7
A365	4734	559	0	0.84	66	0.0001956	38.9	0.001255	290	0.0465	290	0.13	1.3	0.5	1.3	3.7
A366	387	568	0	1.21	29.8	0.0001676	10.6	0.001073	14	0.0465	8.7	0.77	1.1	0.1	1.1	0.1
A367	3440	410	0	0.74	59.9	0.000165	44.3	0.0009012	60	0.0396	40	0.74	1.1	0.5	0.9	0.5
A368	3930	2171	0	2.58	60.5	0.0001651	10.1	0.001051	12	0.0462	6.7	0.83	1.1	0.1	1.1	0.1
A369	4052	236	0	1.44	67.3	0.001175	17.4	0.007916	20	0.0489	9.2	0.88	7.6	1.3	8	1.6
A370	59	688	0	1.27	3.3	0.000149	7	0.0009543	9	0.0465	6.3	0.75	1	0.1	1	0.1
A371	1238	1133	0	1.6	64.9	0.0001551	10.3	0.00128	49	0.0599	48	0.21	1	0.1	1.3	0.6
A372	1008	2017	0	5.06	54.5	0.0001491	7.8	0.001352	55	0.0658	55	0.14	1	0.1	1.4	0.8
A373	49840	1666	1	2.16	66.8	0.0008061	31.8	0.004954	33	0.0446	8.8	0.96	5.2	1.6	5	1.7
A374	30687	1050	72	0.81	0	0.0691	1.8	0.5438	2	0.0571	0.99	0.87	431	7	441	7
A375	1947	1191	0	1.06	18.7	0.0001713	8.4	0.001099	9	0.0466	4.2	0.89	1.1	0.1	1.1	0.1
A377	593	319	0	1.17	48.4	0.0001833	14	0.00118	17	0.0467	8.8	0.85	1.2	0.2	1.2	0.2
A378	60	313	0	1.39	10.7	0.0001699	12.4	0.00109	15	0.0465	8.4	0.83	1.1	0.1	1.1	0.2
A379	100	678	0	1.23	13.5	0.0001417	10.4	0.0008914	13	0.0457	7.8	0.8	0.9	0.1	0.9	0.1
A380	11611	550	13	0.77	9.2	0.02486	1.8	0.168	10	0.049	10	0.18	158	3	158	15
A381	573	555	0	1.16	42.1	0.0001694	9.9	0.001066	13	0.0457	8.5	0.76	1.1	0.1	1.1	0.1
A382	87	629	0	1.25	6.8	0.0001728	7.3	0.001093	10	0.0459	6.4	0.75	1.1	0.1	1.1	0.1
A388	239	491	0	1.07	26.4	0.0001604	8.3	0.0009806	11	0.0444	7.7	0.73	1	0.1	1	0.1
A389	2791	2363	0	1.69	51.3	0.0001462	10.3	0.0009547	12	0.0474	6.5	0.85	0.9	0.1	1	0.1
A390	3009	603	0	1.1	52.1	0.0001611	18.2	0.001017	45	0.0458	41	0.41	1	0.2	1	0.5
A391	17932	2341	1	4	67	0.0003433	25.4	0.002308	27	0.0488	7.5	0.96	2.2	0.6	2.3	0.6
A392	529	671	0	1.11	40.7	0.0001514	12.4	0.0009611	15	0.0461	8.9	0.81	1	0.1	1	0.1
A393	46	295	0	1.29	10.2	0.0001456	11.7	0.0009179	14	0.0457	8.5	0.81	0.9	0.1	0.9	0.1
A394	1802	1084	0	0.97	26.7	0.0001564	12	0.0009985	14	0.0463	7.8	0.84	1	0.1	1	0.1
A395	12283	665	27	0.97	2.1	0.04135	1.7	0.3214	4	0.0564	3.8	0.4	261	4	283	10
A396	135	667	0	1.13	15.6	0.0001396	12.6	0.0008633	15	0.0449	7.5	0.86	0.9	0.1	0.9	0.1
A397	87	749	0	1.33	10.2	0.0001385	9.8	0.0008819	12	0.0462	7	0.81	0.9	0.1	0.9	0.1
A398	4311	376	0	1.05	66.3	0.0008027	14.5	0.005101	17	0.0461	8.1	0.87	5.2	0.7	5.2	0.9
A399	579	1146	0	0.77	0	0.0001677	12.7	0.001063	15	0.046	8.4	0.83	1.1	0.1	1.1	0.2
A400	1585	1057	0	0.64	12.1	0.0001705	11.1	0.001127	14	0.0479	7.8	0.82	1.1	0.1	1.1	0.2
A401	3148	1010	0	0.51	45	0.0001535	15.8	0.001046	31	0.0494	26	0.51	1	0.2	1.1	0.3
A402	1172	1260	0	1.89	44.4	0.0001458	10.3	0.0009177	13	0.0457	7.9	0.79	0.9	0.1	0.9	0.1
A403	148	607	0	1.28	13.8	0.0001732	7	0.001076	10	0.0451	7.5	0.68	1.1	0.1	1.1	0.1
A404	5462	678	9	0.99	6.7	0.01396	2	0.1345	9	0.0699	9.2	0.21	89	2	128	11
A405	2192	1114	0	0.61	41.2	0.0001805	9.3	0.001411	35	0.0567	34	0.27	1.2	0.1	1.4	0.5
A406	63	631	0	1.13	8.5	0.0001428	8.5	0.0009055	12	0.046	7.8	0.74	0.9	0.1	0.9	0.1
A407	4280	1646	0	0.77	43.6	0.0001712	11.7	0.001107	14	0.0469	7	0.86	1.1	0.1	1.1	0.2

References:

1 Frei, D. & Gerdes, A. Precise and accurate in situ U–Pb dating of zircon with high sample throughput by automated LA-SF-ICP-MS. *Chemical Geology* 261, 261-270 (2009).

2 Kohl, C. & Nishiizumi, K. Chemical isolation of quartz for measurement of in-situ-produced cosmogenic nuclides. *Geochimica et Cosmochimica Acta* 56, 3583-3587 (1992).

3 Binnie, S. A. *et al.* Separation of Be and Al for AMS using single-step column chromatography. *Nuclear Instruments and Methods in Physics Research Section B: Beam Interactions with Materials and Atoms* (2015).

4 Dewald, A. *et al.* CologneAMS, a dedicated center for accelerator mass spectrometry in Germany. *Nuclear Instruments & Methods in Physics Research Section B-Beam Interactions with Materials and Atoms* 294, 18-23, doi:10.1016/j.nimb.2012.04.030 (2013).

5 Nishiizumi, K. *et al.* Absolute calibration of Be-10 AMS standards. *Nucl. Instr. Meth. Phys. Res. B* 258, 403-413 (2007).

6 Vermeesch, P. *et al.* Interlaboratory comparison of cosmogenic Ne-21 in quartz. *Quaternary Geochronology* 26, 20-28, doi:10.1016/j.quageo.2012.11.009 (2015).

7 Ma, Y. & Stuart, F. M. The use of in-situ cosmogenic 21 Ne in studies on long-term landscape development. *Acta Geochimica*, 1-13 (2017).

8 Codilean, A. T. *et al.* Single-grain cosmogenic ^{21}Ne concentrations in fluvial sediments reveal spatially variable erosion rates. *Geology* 36, 159-162 (2008).

9 Lifton, N., Sato, T. & Dunai, T. J. Scaling in situ cosmogenic nuclide production rates using analytical approximations to atmospheric cosmic-ray fluxes. *Earth and Planetary Science Letters* 386, 149-160, doi:10.1016/j.epsl.2013.10.052 (2014).

10 Balco, G., Stone, J. O., Lifton, N. A. & Dunai, T. J. A complete and easily accessible means of calculating surface exposure ages or erosion rates from (10)Be and (26)Al measurements. *Quaternary Geochronology* 3, 174-195, doi:10.1016/j.quageo.2007.12.001 (2008).

11 Borchers, B. *et al.* Geological calibration of spallation production rates in the CRONUS-Earth project. *Quaternary Geochronology* 31, 188-198 (2016).

12 Kober, F., Alfimov, V., Ivy-Ochs, S., Kubik, P. W. & Wieler, R. The cosmogenic (NE)-N-21 production rate in quartz evaluated on a large set of existing Ne-21-Be-10 data. *Earth and Planetary Science Letters* 302, 163-171, doi:10.1016/j.epsl.2010.12.008 (2011).

13 Dunai, T. J., González López, G. A. & Juez-Larré, J. Oligocene–Miocene age of aridity in the Atacama Desert revealed by exposure dating of erosion-sensitive landforms. *Geology* 33, 321, doi:10.1130/g21184.1 (2005).

14 Evenstar, L. A., Stuart, F. M., Hartley, A. J. & Tattitch, B. Slow Cenozoic uplift of the western Andean Cordillera indicated by cosmogenic 3He in alluvial boulders from the Pacific Planation Surface. *Geophysical Research Letters* 42, 8448-8455 (2015).

15 Salisbury, M. J. *et al.* 40Ar/39Ar chronostratigraphy of Altiplano-Puna volcanic complex ignimbrites reveals the development of a major magmatic province. *Geological Society of America Bulletin*, B30280. 30281 (2010).

16 Kern, J. M. *et al.* Geochronological imaging of an episodically constructed subvolcanic batholith: U-Pb in zircon chronochemistry of the Altiplano-Puna Volcanic Complex of the Central Andes. *Geosphere* 12, 1054-1077 (2016).

17 Sáez, A., Cabrera, L., Jensen, A. & Chong, G. Late Neogene lacustrine record and palaeogeography in the Quillagua–Llamara basin, Central Andean fore-arc (northern Chile). *Palaeogeography, Palaeoclimatology, Palaeoecology* 151, 5-37 (1999).

18 Sáez, A. *et al.* The stratigraphic record of changing hyperaridity in the Atacama desert over the last 10Ma. *Earth and Planetary Science Letters* 355-356, 32-38, doi:10.1016/j.epsl.2012.08.029 (2012).

19 Gerdes, A. & Zeh, A. Zircon formation versus zircon alteration—new insights from combined U–Pb and Lu–Hf in-situ LA-ICP-MS analyses, and consequences for the interpretation of Archean zircon from the Central Zone of the Limpopo Belt. *Chemical Geology* 261, 230-243 (2009).
